# Glycaemic control and its related factors among people with type 2 diabetes in low- and middle-income countries: a systematic review and meta-analysis

**DOI:** 10.3389/fcdhc.2025.1695235

**Published:** 2025-11-27

**Authors:** Bodrun Naher Siddiquea, Dianna J. Magliano, Hasina Akhter Chowdhury, Mohammad Rocky Khan Chowdhury, Afsana Afroz, Sonali S. Shah, Baki Billah

**Affiliations:** 1 Department of Epidemiology and Preventive Medicine, School of Public Health and Preventive Medicine, Monash University, Melbourne, VIC, Australia; 2 Prothikrit Institute of Health Studies (PIHS), Dhaka, Bangladesh; 3 Diabetes and Population Health, School of Public Health and Preventive Medicine, Monash University, Melbourne, VIC, Australia; 4 Baker Heart and Diabetes Institute, Melbourne, VIC, Australia; 5 Centre for Injury Prevention and Research, Bangladesh (CIPRB), Dhaka, Bangladesh; 6 Department of Population Science, Jatiya Kabi Kazi Nazrul Islam University, Department of Public Health, First Capital University of Bangladesh, Chuadanga, Bangladesh; 7 Obstetrics and Gynaecology, Faculty of Medicine, Dentistry and Health Sciences, The University of, Melbourne, VIC, Australia; 8 Department of Molecular and Translational Science, Monash University, Melbourne, VIC, Australia

**Keywords:** systematic review, meta-analysis, prevalence, factors, glycaemic control, type 2 diabetes mellitus, low- and middle-income countries

## Abstract

**Objective:**

To estimate the prevalence of inadequate glycaemic control and identify factors associated with it among people with type 2 diabetes mellitus (T2DM) living in low- and middle-income countries (LMICs).

**Methods:**

A systematic literature search was conducted in the Medline, Embase, CINAHL, PsychINFO, and Global Health databases for articles published between 1 January 2001 and 15 April 2025. Information was descriptively summarised following the Preferred Reporting Items for Systematic Reviews and Meta-analyses guidelines. The quality of the articles was assessed using the Newcastle-Ottawa Scale. Random effects model was used to obtain the pooled proportion of inadequate glycaemic control. Heterogeneity (I^2^) was tested, sensitivity analyses were performed, and publication bias was examined using Egger’s regression test.

**Results:**

Among 12,985 records, 62 studies from 28 countries involving 176,349 participants were reviewed. The estimated pooled proportion of inadequate glycaemic control (glycosylated haemoglobin [HbA1c] ≥7%) was 69% (95% confidence interval [CI]: 66%–72%, p <0.001, I^2^ = 99.10%), with no publication bias (Egger’s test, p = 0.489). A number of factors were associated with inadequate glycaemic control (overall p < 0.001), including education below secondary level (OR: 1.47, 95% CI: 0.98–1.97), rural residence (OR: 1.80, 95% CI: 1.33–2.28), obesity (OR: 1.17, 95% CI: 1.11–1.22), use of oral glucose-lowering drugs and/or insulin (OR: 4.06, 95% CI: 2.58–5.54 and OR: 2.44, 95% CI: 1.70–3.19, respectively), non-adherence to diet (OR: 2.13, 95% CI: 1.33–2.93) and treatment (OR: 2.08, 95% CI: 1.61–2.54), and physical inactivity (OR: 2.15, 95% CI: 1.35–2.95).

**Conclusion:**

More than two-thirds of people with T2DM in LMICs have inadequate glycaemic control. Urgent interventions are needed, focusing on sociodemographic, lifestyle, and treatment-related factors.

**Systematic Review Registration:**

https://www.crd.york.ac.uk/PROSPERO, identifier CRD: 42023390577.

## 1 Introduction

Type 2 diabetes mellitus (T2DM) is a major global public health challenge with an increasing burden, particularly in resource-limited settings. In 2024, an estimated 589 million adults were living with diabetes worldwide, with approximately 80% residing in low- and middle-income countries (LMICs) (1–3). The number of people living with diabetes is projected to reach 853 million by 2050, with LMICs accounting for 95% of this increase, reflecting a growing disparity in disease burden (1–3). According to the World Bank classification (2024), low income countries have a gross national income (GNI) per capita of USD 1,145 or less, lower-middle-income countries range between USD 1,146 and 4,515, and upper-middle-income countries range between USD 4,516 and 14,005 (4). These countries face significant challenges in diabetes prevention and management, including limited healthcare infrastructure, restricted access to diagnostic testing such as glycosylated haemoglobin (HbA1c), and inconsistent long-term follow-up care.

T2DM substantially increases the risk of major macrovascular (heart disease, stroke, lower-limb amputation) and microvascular (retinopathy, nephropathy, neuropathy) complications, which contribute to disability and premature mortality (5). Maintaining adequate glycaemic control, most commonly assessed by HbA1c, is a cornerstone of diabetes management and is strongly linked to a reduced risk of complications and improved quality of life. The American Diabetes Association recommends an HbA1c target of <7% (6), yet globally only 50% of people with diabetes achieve this target. Control rates are markedly lower in LMICs (37%) compared with high-income countries (52.2–53.6%) (7–9).

Multiple interrelated factors influence glycaemic control. Individual-level determinants include obesity, physical inactivity, unhealthy dietary patterns, and medication non-adherence (10–12), while sociodemographic factors such as lower educational attainment and rural residence, and low socioeconomic status are associated with suboptimal control (12). Health system barriers including limited access to healthcare, fragmented care continuity, and medication shortages further exacerbate poor outcomes (12). Moreover, cultural and lifestyle factors, such as dietary patterns and healthcare-seeking behaviours, differ significantly from those in high-income countries, influencing diabetes outcomes in unique ways (13).

Although several reviews have examined glycaemic control, most have included both type 1 and type 2 diabetes or focused on specific regions such as sub-Saharan Africa or the Gulf countries (10, 12–14). Consequently, available evidence remains fragmented and highly context-specific, limiting the generalisability of findings across LMICs. Understanding the magnitude and determinants of inadequate glycaemic control in these settings is crucial for developing targeted, context-appropriate interventions and informing national and global policy to improve diabetes outcomes in resource-limited environments.

The rationale for this systematic review and meta-analysis is therefore stems from the global rise in T2DM, its disproportionate impact on LMICs, and the lack of comprehensive synthesis of existing evidence. Hence, this review aims to estimate the prevalence of inadequate glycaemic control (HbA1c ≥7%) and identify its associated factors among adults with T2DM in LMICs. By integrating data from diverse settings, this study seeks to provide a more complete understanding of the determinants of inadequate glycaemic control and to highlight priority areas for targeted intervention.

## 2 Methods

**Figure 1:**
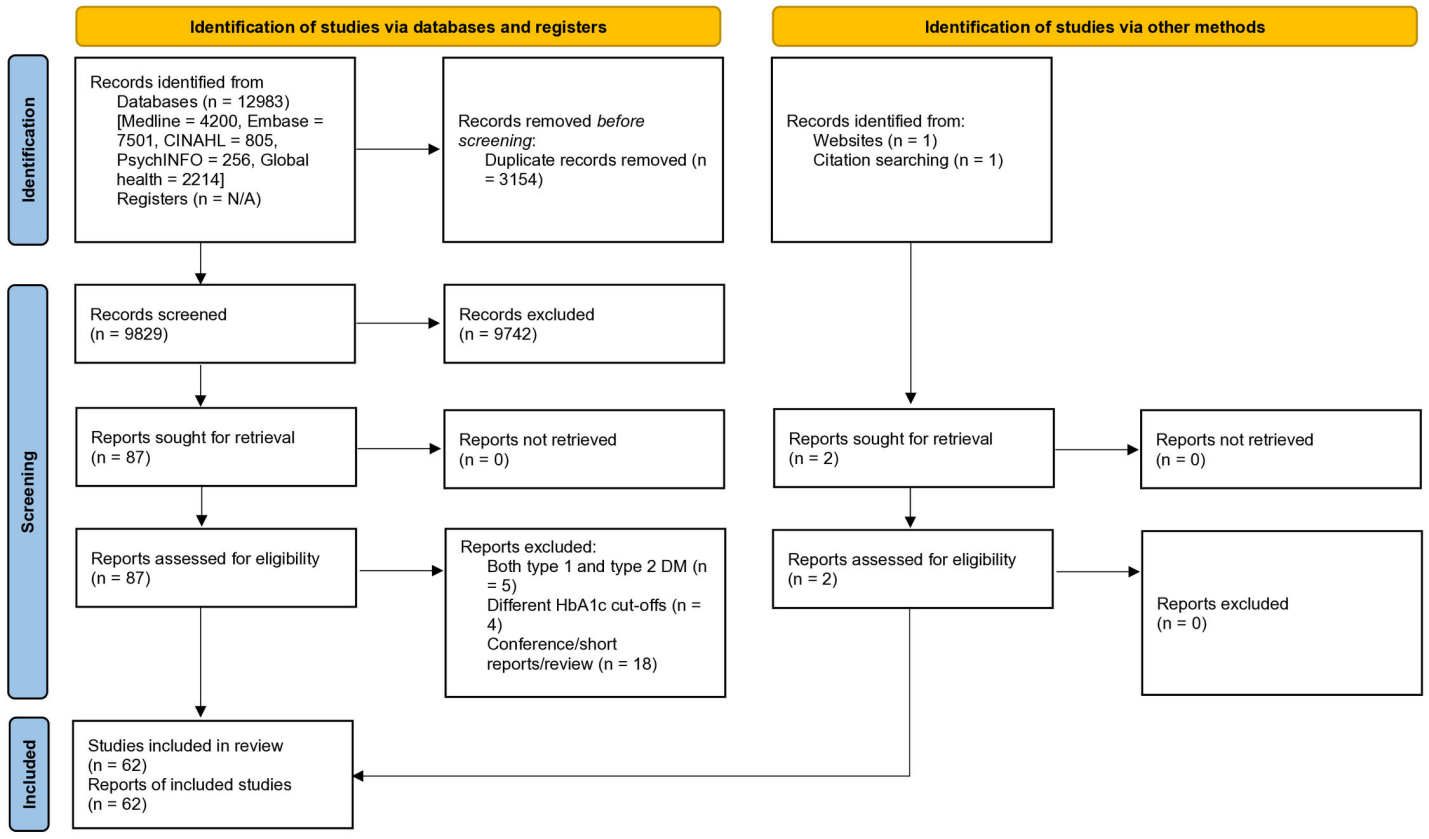
Preferred reporting items for systematic reviews and meta-analyses (PRISMA) 2020 flow diagram.

This systematic review and meta-analysis was registered with PROSPERO (CRD: 42023390577) and conducted according to the Preferred Reporting Items for Systematic Reviews and Meta-analyses (PRISMA) guidelines (**Appendix 1**) (15). The review process is illustrated in **Figure 1**.

### 2.1 Selection criteria

This review followed the PECO framework: P (population), E (Exposure), C (comparison), O (outcome). Population – adults (≥18 years) with type 2 diabetes mellitus living in low- and middle-income countries; Exposure – sociodemographic, lifestyle, and clinical factors potentially associated with glycaemic control; and Outcome – inadequate glycaemic control, defined as HbA1c ≥7%.

All published observational studies (cross-sectional, case–control, and retrospective or prospective cohort designs) reporting the prevalence of inadequate glycaemic control, assessed by HbA1c testing, among adults with T2DM in LMICs were eligible for inclusion. There were no restrictions on language or publication year.

Studies were excluded if they:

1. Focused exclusively on individuals with type 1 diabetes, both type 1 and type 2 diabetes, gestational diabetes, or diabetes-related complications;
2. Did not define or report glycaemic control, or used non-standard HbA1c thresholds or mean HbA1c values instead of categorical definitions;
3. Were qualitative studies, randomised controlled trials, or were not primary research (e.g., editorials, commentaries, abstracts, dissertations, reviews, or case reports).

### 2.2 Search strategy

A systematic literature search strategy was developed in consultation with a librarian from Monash University. The search focused on a range of keywords relating to glycaemic control, T2DM and a list of LMICs based on the current World Bank Database (4). Five databases (Medline, Embase, CINAHL, PsychINFO and Global Health) were searched between 1 January 2001 and 15 April 2025 by two authors (BNS and HAC) independently, following the developed search strategy. The detailed search strategy is provided in **Appendix 2**. Additionally, reference lists of the included studies and websites (e.g., Google Scholar) were manually searched to identify potential articles.

### 2.3 Study selection process

Searched articles were stored and managed using the citation software EndNote X20. After the searches, BNS and HAC independently removed the duplicates. Furthermore, titles and abstracts were screened independently for inclusion in the review. The authors also independently reviewed the full text of the remaining articles. Any discrepancies between reviewers were addressed by consultation with the senior author (BB). Finally, 62 articles were included in this review.

### 2.4 Study outcome and exposures

The outcome of this review was the prevalence of people with T2DM who had inadequate glycaemic control (expressed as a percentage), defined as an HbA1c level of ≥7% (6). The exposures included any factors associated with inadequate glycaemic control (e.g., socio-demographic, clinical, anthropometric, behavioural, and psychological), reported as odds ratios.

### 2.5 Data extraction

Two authors (BNS and MRKC) independently extracted data from the included articles using Microsoft Excel. Another author (HAC) then cross-checked the extracted data. Any discrepancies were discussed and resolved between the authors, and the senior author BB made the final recommendation where necessary. The following information was extracted: publication details (authors, year of publication, journal), study characteristics (country where the study was conducted, study design, study setting, study population, sample size), participants’ demographics (gender, age, education, occupation) and the prevalence of poor/inadequate or good/adequate glycaemic control and factors associated with it. Missing data were sought from the authors of the studies, where required.

### 2.6 Quality assessment

The quality of all included studies was independently assessed by two authors (BNS and HAC) using the Newcastle-Ottawa Scale (NOS) for observational studies (cross-sectional, case-control and cohort studies) (16). The tool utilises a ‘star system’ to evaluate studies from three primary perspectives: the selection of study groups, the comparability of those groups, and the identification of the exposure of interest (outcome) for cross-sectional and cohort studies. Cut-off values of 0-4, 5–6 and ≥7 were used to classify the studies as poor, fair and good quality, respectively. Discrepancies were resolved by the senior author BB. A detailed description of the quality assessment tool is provided as supporting information (**Appendix 3**).

### 2.7 Data analysis

Extracted data were analysed (BNS) and cross-checked by another author (AA). A random-effects model was used to estimate the pooled prevalence of inadequate glycaemic control and other relevant quantitative data with a 95% confidence interval (CI). Heterogeneity among studies was tested using the χ^2^ test on Cochran’s *Q* statistic, which was calculated using H and I^2^ indices. Along with a non-significant result (p-value >0.05), an I² value >50% was considered to indicate substantial heterogeneity, while a value >75% was considered considerable heterogeneity (17). An individual study contribution to overall heterogeneity was assessed by excluding each study and recording the change in overall heterogeneity (18). Subgroup/sensitivity analyses were also performed with the covariates such as participants’ sociodemographic, clinical, and behavioural characteristics, as well as study quality, geographic region, and the income level of the country, to identify possible substantial/considerable heterogeneity. The income level of each country was classified as low-income, lower-middle-income and upper-middle-income according to the World Bank (4). Publication bias was examined by generating funnel plots and quantitatively by Egger’s regression test (19). All the statistical analyses were conducted using Stata V.16 (StataCorp, College Station, Texas, USA).

## 3 Results

A total of 12,985 articles, published between 1 January 2001 and 15 April 2025, were retrieved. These articles were sourced from five databases and supplemented with manual searches. After the removal of duplicate records and screening based on titles and abstracts, a refined selection of 89 articles was deemed suitable for full-text review. From this refined pool, 27 articles were excluded due to non-compliance with the inclusion criteria. Eventually, a dataset of 62 studies, spanning 28 different countries, was identified (**Figure 1**) (20–81). These studies collectively represented a participant pool of 176,349 individuals (the study’s sample size ranged from 92 to 55,639).

### 3.1 Characteristics of the study participants

All included studies were cross-sectional, except for one retrospective cohort survey. Approximately half were conducted in hospitals (diabetes clinics/centres) (n = 33; 53%), followed by primary care centres (n = 23; 38%) and hospital in-patient settings (n = 2; 3%). Only four were community-based studies (6%). All of the studies included people with T2DM except one which included people with type 1 and those with T2DM but reported results separately. In this review, only data on T2DM were included in the analysis. Most of the studies (57 out 62) were published in the year 2010 or later. Of the included studies, 31 (50%) were conducted in upper-middle- income countries, 26 (42%) in lower-middle-income countries and only five (8%) in low-income countries. By geographic region, 38 (61%) studies were from Asia, 19 (31%) studies were from Africa and the remaining five (8%) were from South America. Gender was reported in the majority of the studies with 54.3% of participants across the combined sample being female. The age of the study participants ranged from 18 to 93 years. Out of 62 studies, 44 studies reported participants’ educational attainment; only 21.7% of participants had continued beyond secondary school. Employment status was reported in 32 studies and 34.7% of participants were employed. **Table 1** shows the detailed characteristics of the included studies. No studies addressed the handling of missing information. Multiple logistic regression was used to identify factors related to glycaemic control. Although machine learning algorithms are emerging as an efficient method for determining factors related to health outcomes, none of the studies included in this review utilised them.

**Table 1:** Characteristics of the studies included in the systematic review and meta-analysis.

Study, year	Study location	Study design	Study period	Study setting	Participants	Age (mean ± SD)/range, years	DM duration (mean ± SD)/median, years	Sample	Female, %	Inadequate glycaemic control	
										n	%
Abd-Elraouf, 2020 (20)	Egypt	Cross-sectional study	July to December, 2019	University hospital (in-patients)	Type 2 diabetes mellitus patients who were admitted to the Internal Medicine Department	NR	NR	200	57.0	186	93.0
Abdullah et al, 2019 (21)	Malaysia	Cross-sectional study	August to November 2017	Two government health clinics	T2DM patients aged 18 years and above	28-90	6.8 ± 4.8	200	59.0	132	66.0
Abera et al, 2022 (22)	Ethiopia	Cross-sectional study	March 1 to May 30, 2021	Diabetic clinic	Type 2 DM attending outpatient medical diabetic clinic of Tikur Anbessa Specialized Hospital	45-62	9	325	57.2	240	73.8
Adeniyi et al, 2016 (23)	South Africa	Cross-sectional study	June to November 2013	15 community health centres (primary care centres)	Patients with T2DM aged ≥ 30 years	NR	NR	327	70.3	274	83.8
Afroz et al, 2019 (24)	Bangladesh	Cross-sectional study	March to September 2017	Six diabetes hospitals	Type 2 DM patients aged ≥18 years old and diabetes duration for 1 year	54.1 ± 12.1	9.9 ± 7.2	1253	57.5	1071	81.8
Ahmad et al, 2014 (25)	Malaysia	Cross-sectional survey	January to July 2008	Primary Health Clinics	Type 2 diabetes mellitus aged ≥ 20 years	55.9 ± 9.1	NR	557	63.2	419	77.0
Akter, 2020 (26)	Bangladesh	Cross-sectional study	July 2018 to June 2019	Endocrinology outpatient clinic	Patients with T2DM	46.2 ± 9.5	4.62 ± 3.76	486	65.8	225	46.3
Al-Zurfi et al, 2012 (27)	Malaysia	Cross-sectional study	February 2010	Diabetic clinic	Type 2 Diabetes Mellitus, Malay and adults aged 18 years old	59.7 ± 9.04	9.0 ± 9.0	190	55.3	170	89.5
Amsah et al, 2022 (28)	Malaysia	Cross-sectional survey	December 2020 to May 2021	10 primary health-care facilities (database)	Patients aged ≥18 years old with of T2DM	NR	NR	3100	73.9	1834	59.2
Artha et al, 2019 (29)	Indonesia	Retrospective cross-sectional	2018-2019	Hospital database	Patients with T2DM	30-65	NR	140	55.0	56	40.0
Ashraf et al, 2020 (30)	India	Retrospective cross-sectional	January 2018 to December 2019	Endocrine super-speciality clinics (database)	Patients with T2DM	49.7 ± 11.3	3.9 ± 4.6	12140	49.5	8829	72.7
Ashur et al, 2016 (69)	Libya	Cross-sectional study	October 2013 to December 2013	Outpatient clinics of Diabetes centre	Patients with T2DM	54.4 ± 10.0	9.4 ± 97.3	523	59.6	409	77.2
Babaniamansour et al, 2020 (31)	Iran	Cross-sectional study	2017-2018	Endocrinology clinic of teaching hospitals	Type 2 DM patients aged 18 years and above	56.06 ± 10.4	8.9 ± 7.1	562	64.4	432	76.9
BeLue et al, 2016 (32)	Senegal	Cross-sectional study	June 2013	Hospital (interview)	Patients with T2DM	55.2 ± 11.6	NR	106	73.6	80	75.5
Bi et al, 2010 (33)	China	Cross-sectional survey	July 20 to 31, 2009	Diabetes centres in 15 primary hospitals, 18 secondary hospitals, and 23 tertiary hospitals	Type 2 DM patients for >6 months	56.4 ± 11.2	6.3 ± 5.7	2966	50.8	1302	43.9
Borgharkar and Das, 2019 (34)	India	Retrospective cross-sectional	2015-2017	Urban healthcare facilities (database)	Patients with type 2 diabetes receiving oral hypoglycaemic agents (OHAs) with or without insulin	54.3 ± 11.1	NR	55639	45.2	42594	76.6
Camara et al, 2014 (35)	Cameroon and Guinea	Cross-sectional study	200-2010	Six regional diabetes management centres in Cameroon and four in Guinea	Patients with T2DM	58.4 ± 10.5	7.6 ± 6.3	1267	61.2	939	74.1
Chetoui et al, 2022 (36)	Morocco	Cross-sectional survey	2017	15 primary health centres	Patients with T2DM for ≥1 year and aged ≥18 years	56.2 ± 11.8	8.63 ± 6.8	1456	73.4	965	66.3
Chua and Chan, 2011 (37)	Malaysia	Cross-sectional study	NR	Diabetes clinic of a tertiary hospital	Type 2 diabetes aged ≥18 years and on antidiabetic medications for at least 3 months	60.3 ± 10.3	NR	405	55.6	336	83.0
Demoz et al, 2019 (38)	Ethiopia	Cross-sectional study	August 2017 to July 2018	Diabetic clinic in a teaching hospital	Patients with T2DM for ≥1 year and aged ≥18 years and were taking at least one antidiabetic drug	56.0 ± 11.0	11.6 ± 6.95	357	52.9	244	68.3
Diaf and Khaled, 2017 (39)	Algeria	Cross-sectional study	March 2013 to June 2014	Public Establishment of Local Health	Patients with T2DM for at least 6 months	55.6 ± 11.0	6.6 ± 3.59	210	65.2	102	48.6
Djonor et al, 2021 (40)	Ghana	Cross-sectional study	May to June, 2018	Greater Accra Regional Hospital	Patients diagnosed with T2DM and on treatment for at least six months	56.6 ± 13.8	NR	271	71.6	161	59.4
Eid et al, 2003 (41)	Malaysia	Cross-sectional study	2001-2002	Diabetes Clinic, Hospital Universiti	Patients with T2DM	NR	NR	211	52.1	153	72.5
Firouzi et al, 2015 (42)	Malaysia	Baseline assessment study	2009	Universiti Kebangsaan Malaysia Medical Centre	Patients with T2DM	56.7 ± 9.9	6.5 ± 5.0	104	61.5	83	79.8
Goyal et al, 2019 (43)	India	Cross-sectional study	January to June, 2018	Retrospective data collected from the diabetic patients who attended the medical outdoor	Patients with type 2 diabetes mellitus attending medicine OPD and >20 years old, receiving antidiabetics medications	56.6 ± 12.4	8.1 ± 6.9	206	44.2	174	84.5
Gumilas et al, 2021 (44)	India	Cross-sectional study	July to December, 2021	Medicine outpatient department of a tertiary care hospital	Type 2 diabetes mellitus patients aged more than 18 years	51.4 ± 14.8	NR	403	31.8	231	57.6
Gurjar et al, 2023 (45)	Indonesia	Cross-sectional study	October, 2018	Primary Health Facility	Patients with T2DM	NR	NR	92	51.1	52	56.5
Hassan et al, 2021 (46)	Malaysia	Cross-sectional study	2014-2018	National Diabetes Registry	Patients with T2DM	61.5 ± 10.9	NR	25062	64.8	18217	72.7
Howteerakul et al, 2007 (47)	Thailand	Cross-sectional study	NR	Diabetes clinic of a tertiary hospital	Patients with T2DM	60.2 ± 9.6	7	243	65.8	162	66.7
Ibrahim et al, 2021 (48)	Nigeria	Retrospective observational study	August to November 2020	Family medicine clinic of a tertiary hospital	Patients with T2DM aged 40 years and above	61.9 ± 11.8	NR	300	58.0	120	40.0
Ismail et al, 2016 (49)	Malaysia	Cross-sectional study	January to December, 2013	Universiti Kebangsaan Malaysia Medical Centre (in-patients)	Patients with T2DM	58.8 ± 12.6	NR	127	43.3	100	78.7
Khattab et al, 2008 (50)	Jordan	Cross-sectional study	2008	National Center for Diabetes, Endocrinology, and Genetics	Patients with Type 2 diabetes aged 18 years or above	57.4 ± 9.6	NR	917	50.4	597	65.1
Kumar and Sandhya, 2017 (51)	India	Cross-sectional study	NR	Diabetes care center	Patients with type 2 diabetes	18-65	NR	1200	49.2	860	71.7
Li et al, 2018 (52)	China	Cross-sectional study	1 July 2012 to 30 June 2017	Medical records’ database	Type 2 DM at least for 6 months and aged 18 years and above	54.1	NR	1387	43.1	698	50.3
Lima et al, 2016 (53)	Brazil	Cross-sectional study	November 2009 and December 2010	Family Health Strategy (integrated primary care)	People with type 2 DM	61.1 ± 13.1	8.6 ± 7.5	787	68.2	545	69.3
Mashele et al, 2019 (56)	South Africa	Cross-sectional study	NR	Diabetic clinic in a tertiary hospital	Patients with type 2 diabetes	58.9 ± 11.5	NR	176	69.9	149	84.7
Mahmood et al, 2016 (54)	Malaysia	Cross-sectional study	January to December, 2013	13 Public health clinics	Patients with T2DM aged 18 years and above	58.7 ± 12.3	6.9 ± 5.1	706	59.1	480	68.0
Maiftrianti et al, 2020 (55)	Indonesia	Cross-sectional study	July to August 2019	Primary health care	Patients with T2DM aged 18 years and above	61.0 ± 9.1	NR	126	70.6	69	54.8
Mendes et al, 2009 (57)	Brazil	Cross-sectional study and nationwide survey	February 2006 to March 2007	Diabetes clinics (outpatient)	Patients with either type 1 and 2 DM aged 18 years and above	NR	NR	5692	33.4	4162	73.1
Mobula et al, 2018 (58)	Ghana	Cross-sectional study	NR	Hospitals	Type 2 DM patients aged 18 years and above	18-89	NR	1226	72.7	858	70.0
Moreira et al, 2010 (59)	Venezuela	Cross-sectional study and nationwide survey	January to June 2007	Diabetic clinics	Patients with T2DM aged ≥18 years	18-93	NR	3726	65.1	2791	74.9
Mwavua et al, 2016 (60)	Kenya	Cross-sectional study	August to October 2012	National Referral Hospital and one primary healthcare facility	Patients with T2DM for 6 months and on glucose lowering therapy for at least 3 months	57.8 ± 12.3	NR	200	66.5	166	83.0
Najeeb et al, 2022 (61)	India	Cross-sectional study	December 2019 to March 2020	Community-based	Patients with T2DM for ≥5 year and aged ≥18 years	60.0 ± 8.9	NR	364	58.5	286	78.6
Nyunt et al, 2010 (64)	Myanmar	Cross-sectional study	February to March, 2009	Two private clinics	Patients with T2DM for ≥1 year and aged ≥35 years	57.5 ± 10.9	5.5	266	57.9	194	72.9
Noor et al, 2017 (63)	Sudan	Cross-sectional hospital-based study	NR	Diabetes centre	Patients with T2DM for ≥1 year and aged ≥18 years	20-75	NR	387	49.6	328	84.8
Omar et al, 2018 (65)	Sudan	Cross-sectional study	February to August 2017	University clinics	Patients with T2DM aged ≥18 years	54.8 ± 12.8	5.8 (3-10)	339	69.9	243	71.7
Patrick et al, 2021 (66)	Uganda	Cross-sectional study	July to October, 2020	Regional Referral Hospital outpatient diabetes clinic	Patients with T2DM for 6 months and aged ≥18 years	61.1 ± 4.0	NR	223	68.6	190	85.2
Rossaneis et al, 2017 (67)	Brazil	Cross-sectional study	2012	38 Basic Health Units	Patients with T2DM for 6 months and aged ≥40 years	NR	NR	746	65.5	521	69.8
Saghir et al, 2019 (68)	Yemen	Cross-sectional study	January to March 2017	Outpatient clinic at the Military Hospital	Patients with T2DM for >1 year	49.5 ± 11.8	NR	246	47.6	180	73.2
Shuhaida et al, 2019 (69)	Malaysia	Cross-sectional study	December 2014 to June 2015	Two health clinics with family medicine specialists	Patients with T2DM for ≥5 year and aged ≥18 years	60.9 ± 10.3	NR	338	62.7	257	76.0
Siddiqui et al, 2014 (70)	Pakistan	Cross-sectional study	September 2002 to January 2003	Outpatient diabetic clinic	Patients with T2DM for 3 months and aged ≥40 years	26-88	5.5 (0.5-35.5)	452	72.1	176	38.9
Soffian et al, 2019 (71)	Malaysia	Cross-sectional study	August 2016 to July 2017	National Diabetes Registry	Patients with T2DM	NR	6.2 ± 7.16	23557	63.4	13136	55.8
Tharek et al, 2018 (73)	Malaysia	Cross-sectional study	August 2014 to September 2015	Two public primary care clinics	Patients with T2DM for ≥1 year and aged ≥18 years	58.3 ± 11.9)	NR	340	58.8	294	86.5
Thaneerat et al, 2009 (72)	Thailand	Cross-sectional study	June to December 2008	Endocrine clinic of hospital	Patients with type 2 DM at least for 1 year	62.6 ± 10.4	12.8 ± 8.4	250	64.4	140	56.0
Thuita, 2019 (74)	Kenya	Cross-sectional study	NR	Hospital Diabetes Comprehensive Care Centre	Patients with type 2 DM aged 20–79 years	20-79	NR	153	59.5	119	77.8
Thuy et al, 2021 (75)	Vietnam	Retrospective Cohort survey	October 2018 to October 2019	Urban hospital	Patients with T2DM	62.4 ± 7.6	7.9 ± 6.4	189	55.6	133	70.4
Ufuoma et al, 2016 (76)	Nigeria	Cross-sectional study	March to August,2014	Diabetes outpatient clinics of Central Hospital Warri	T2DM patients aged 40 years	54.8 ± 11.9	8.5 ± 3.2	200	52.0	111	55.5
Viana et al, 2013 (77)	Brazil	Cross-sectional study	February 2006 and April 2011	Outpatient clinics of hospitals and primary care units	Patients with T2DM diagnosed after 30 years of age without insulin use in the first 5 years after the diagnosis.	61.0 ± 10.0	11.0 ± 8.0	5750	66.0	2959	51.5
Wang et al, 2021 (78)	China	Cross-sectional study	January to December 2018	Su Value database (both in-patients and outpatients)	Patients with T2DM on oral medications	NR	NR	13972	49.5	7819	56.0
Wan et al, 2016 (79)	Malaysia	Cross-sectional study	NR	Five health clinics	Adults with type 2 diabetes mellitus receiving treatment for one year	30-88	7.63 ± 5.3	324	65.1	215	66.4
Xing et al, 2022 (80)	China	Cross-sectional study	August to December 2018	Community-based	Patients with T2DM	18-75	NR	1715	50.7	1321	77.0
Yeemard et al, 2022 (81)	Thailand	Cross-sectional study	February to May, 2021	Diabetic clinics in six hospitals	Patients with type 2 DM at least for 2 year	58.7 ± 11.3	NR	967	58.8	530	54.8

### 3.2 Quality of included studies

The quality of the included studies was assessed using the Newcastle-Ottawa Scale (NOS) for cross-sectional and cohort studies, with ratings ranging from poor to good. Most studies scored well in the selection domain, particularly in terms of the representativeness of the study population, although limitations were noted in the ascertainment of exposure (19 studies). The comparability domain revealed shortcomings, as 10 studies did not adjust for key confounders such as age and sex. Additionally, 10 studies had issues in the outcome domain, particularly related to statistical analysis. Overall, 19 studies were rated as poor quality, 26 as fair, and 17 as good. These quality categories were considered in the sensitivity analysis (**Appendix 4 and 5**).

### 3.3 Glycaemic control (assessment and prevalence)

**Figure 2:**
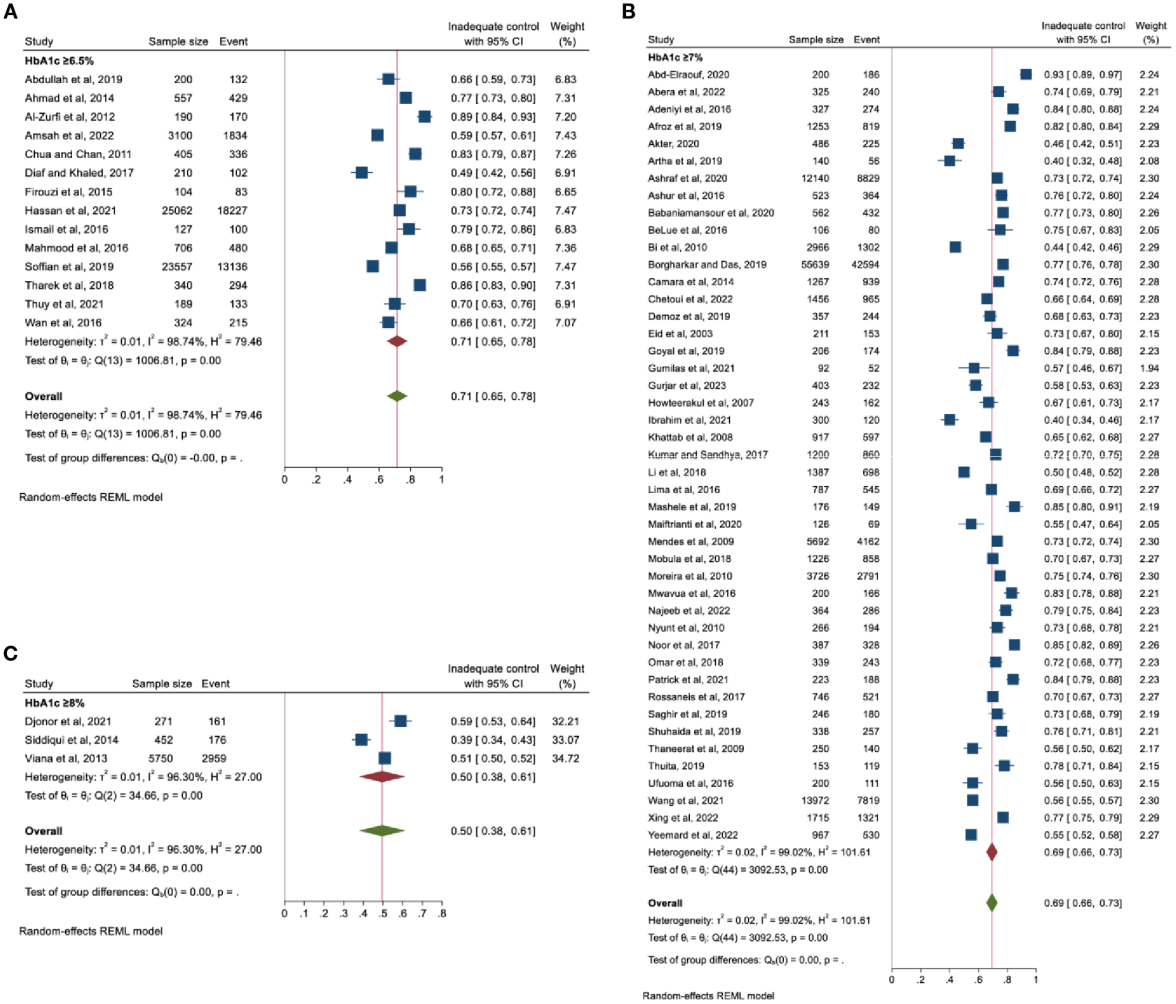
Pooled prevalence of inadequate glycaemic control based on HbA1c thresholds **(A)** ≥6.5%, **(B)** ≥7%, and **(C)** ≥8%.

All the included studies assessed glycaemic control by HbA1c (%), but the cut-offs for inadequate glycaemic control varied: 14 studies used HbA1c ≥6.5%, 45 used HbA1c ≥7% and the remaining three used HbA1c ≥8% (**Figure 2**).

**Figure 3:**
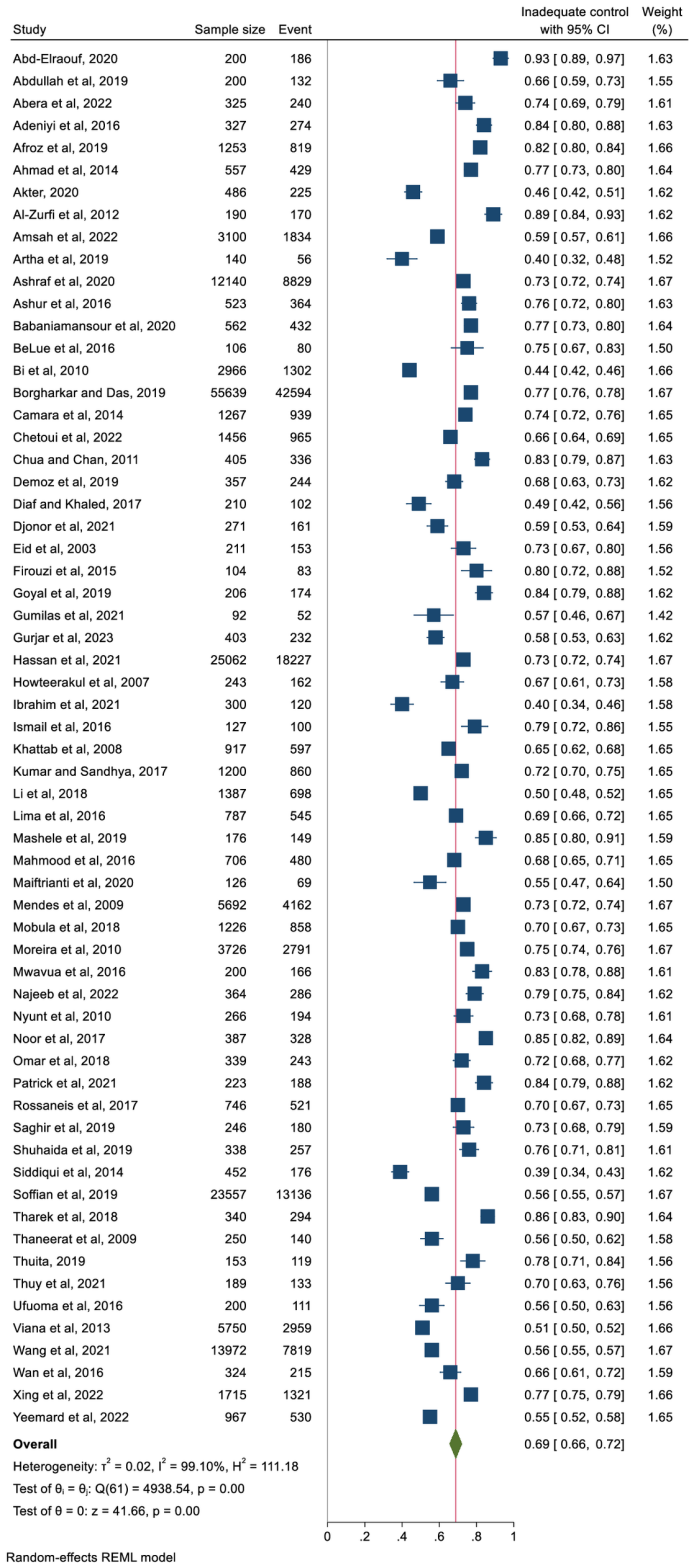
Pooled prevalence of inadequate glycaemic control (defined as HbA1c≥6.5% or ≥7% or ≥8%).

**Figure 3** represents the pooled proportion of inadequate glycaemic control. The findings demonstrated that 69% (n = 127,577) of the study participants had inadequate glycaemic control (95% CI: 66%–72%, p <0.001). The prevalence ranged from 40% (in Nigeria) to 93% (in Egypt). High heterogeneity was observed (I^2^ = 99.10%) with no publication bias (Egger’s regression test, p = 0.489).

**Figure 4:**
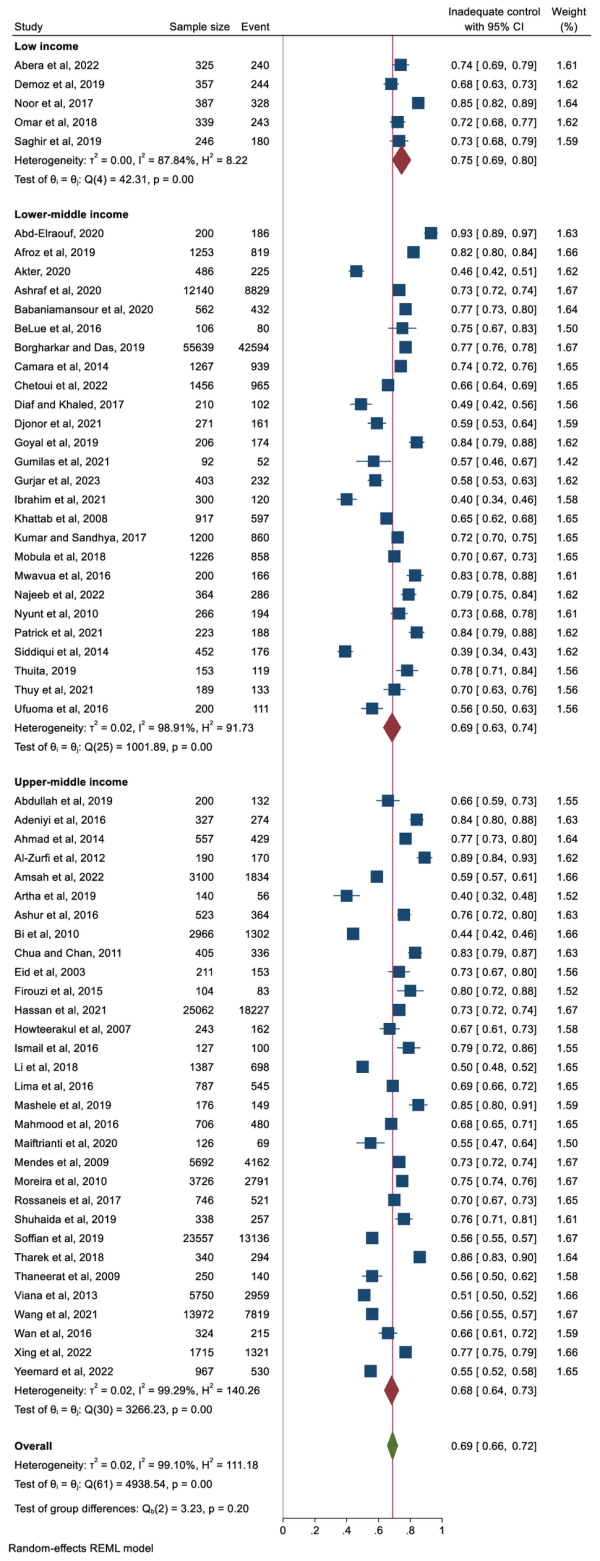
Pooled prevalence of inadequate glycaemic control (defined as HbA1c ≥6.5%, ≥7%, or ≥8%) stratified by country income level.

In terms of the income level of the country, the pooled proportion of participants with inadequate glycaemic was 68% in upper-middle income countries and 69% in lower-middle-income countries and 75% in low-income countries (**Figure 4**). However, the differences were not significant. Analysis by geographic region also showed non-significant differences. The pooled proportion of people with inadequate glycaemic control was 72% (95% CI: 66%–78%) in Africa, 68% (95% CI: 59%–76%) in South America and 67% in Asia (95% CI: 63%–72%). The prevalence of people with inadequate glycaemic control in each country included in this review is presented in **Supplementary Figure 1**.

A trend analysis using linear regression was performed to assess changes in the prevalence of inadequate glycaemic control across study years. The fitted line indicates a slight, non-significant overall decline in prevalence from 2003 to 2023 (p-value = 0.52) (**Supplementary Figure 2**). The prevalence was also stratified by study setting and presented in **Supplementary Figure 3**.

### 3.4 Factors associated with inadequate glycaemic control

**Figure 5:**
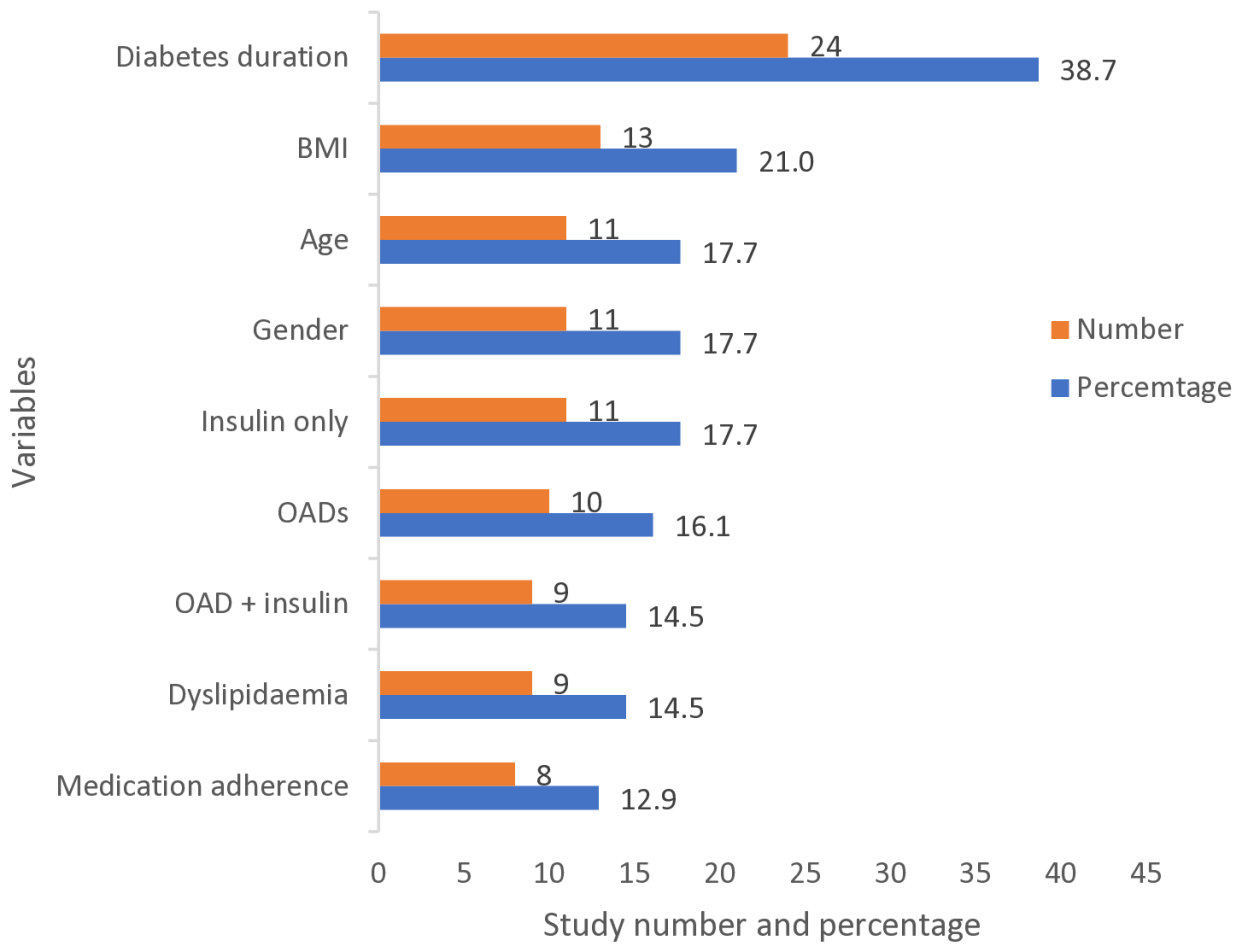
Most frequent factors associated with inadequate glycaemic control (defined as HbA1c ≥6.5%, ≥7%, or ≥8%) reported by the studies. BMI, Body Mass Index; OAD, Oral Anti-diabetic Drug.

**Figure 6:**
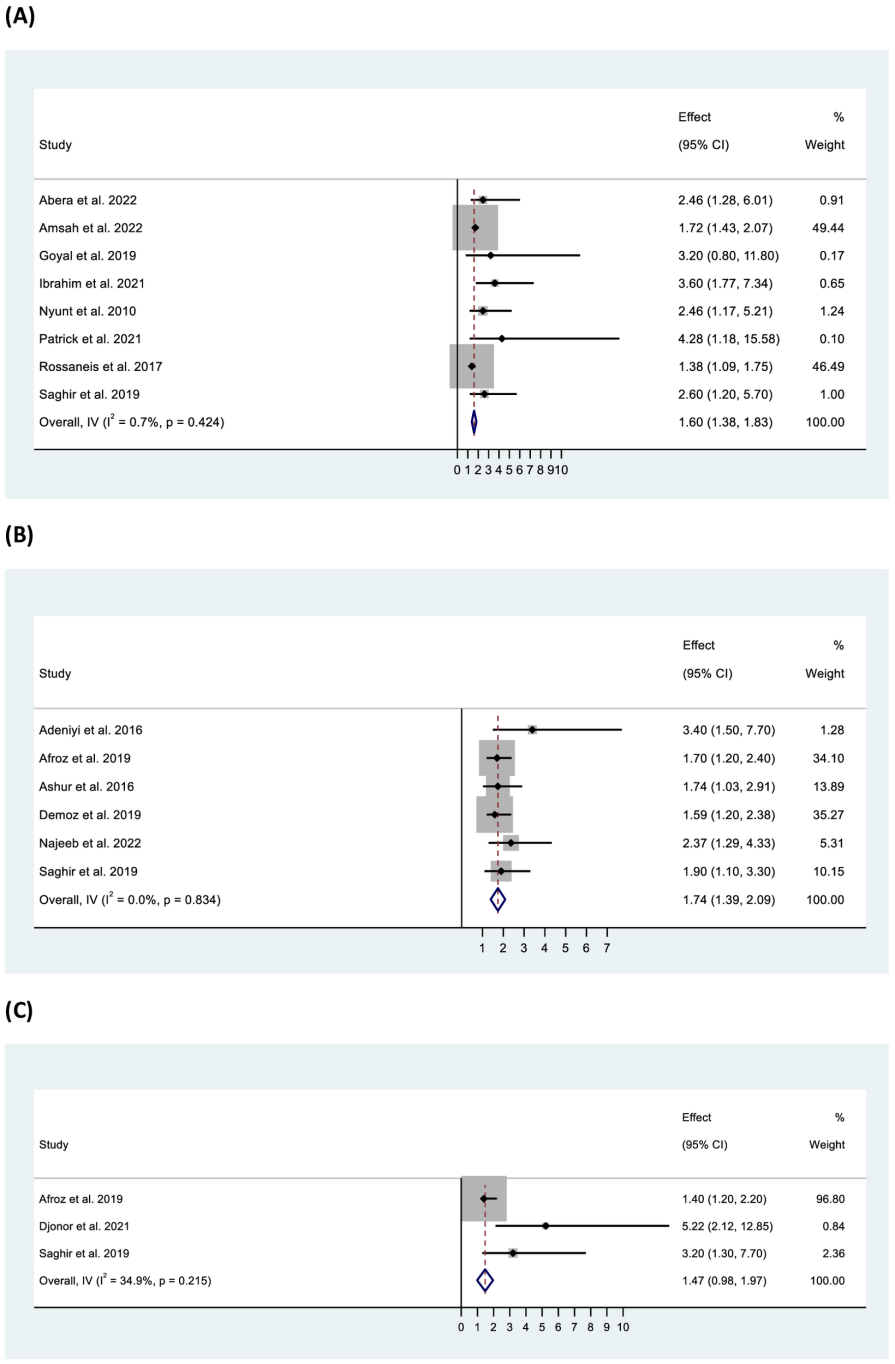
Forest plot of odds ratio of inadequate glycaemic control in relation to **(A)** age (>60 years), **(B)** gender (female), and **(C)** education (below secondary school).

Factors associated with inadequate glycaemic control reported in the included studies are presented in the supporting information (**Appendix 6**). A total of 35 factors were reported by these studies. Seven factors – longer diabetes duration, older age, overweight/obesity, diabetes treatment modalities (oral antidiabetic drugs [OADs] and/or insulin), female gender, medication non-adherence, and the presence of dyslipidaemia – were reported by ≥10% of the studies (**Figure 5**). Other factors, including education below secondary, rural residence, presence of hypertension, high waist circumference, physical inactivity, dietary non-adherence and presence of micro- and macrovascular complications were reported by <10% of the studies. Overall pooled data from eight studies showed a significant association between inadequate glycaemic control and older age (>60 years) (OR: 1.60, 95% CI: 1.38–1.83, p < 0.001), whereas three studies found inadequate control among participants aged <60 years (OR: 2.06, 95% CI: 1.51–2.62, p < 0.001). Women had higher odds than men of inadequate glycaemic control (OR: 1.74, 95% CI: 1.39–2.09, p < 0.001). Analysis showed significantly higher odds of inadequate control in participants with an education level below secondary, on lower income and residing in rural areas. The studies were homogenous in all of the above analyses except for the analysis related to education which showed low to moderate heterogeneity (**Figure 6**).

**Figure 7:**
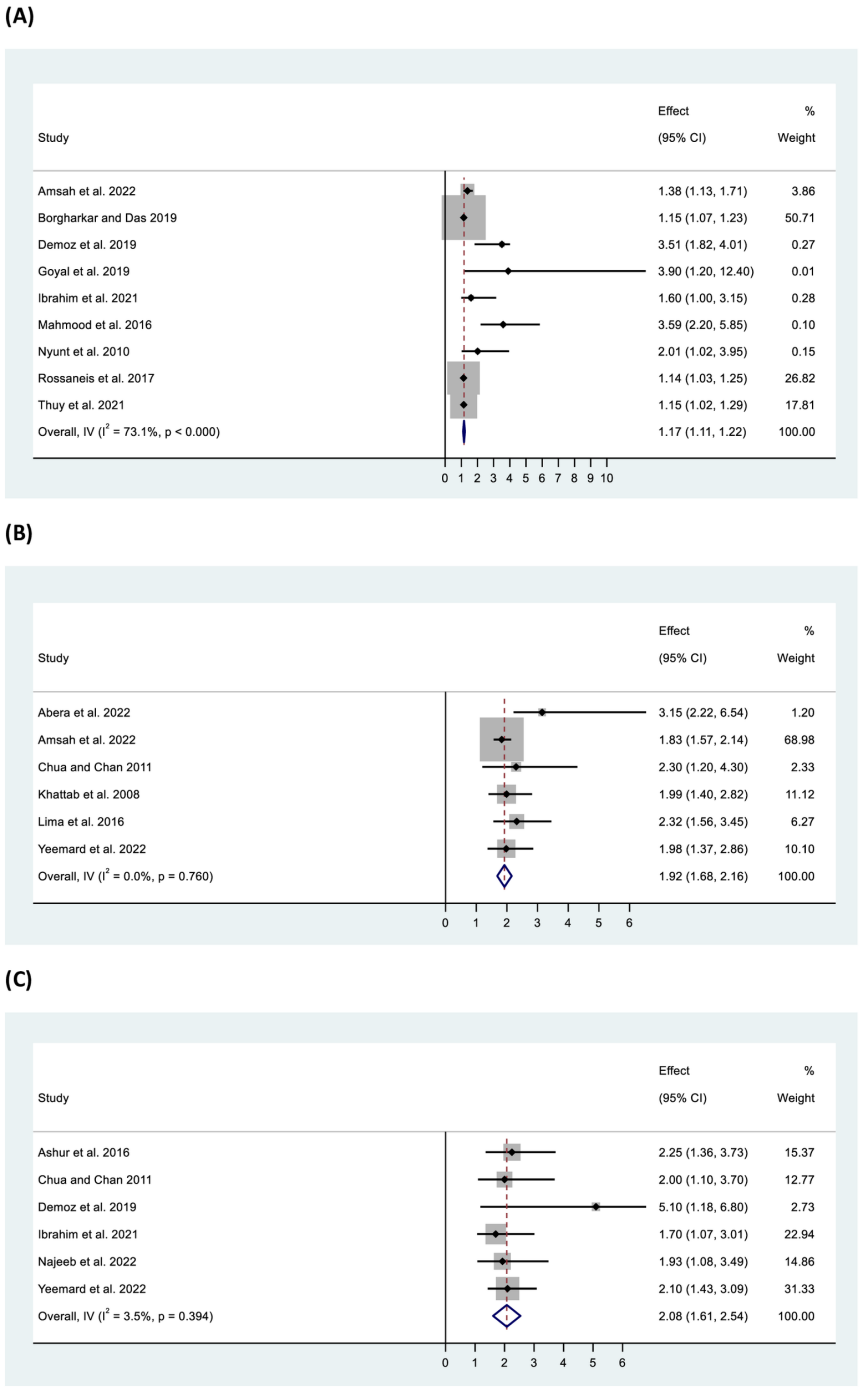
Forest plot of odds ratio of inadequate glycaemic control in relation to **(A)** BMI (overweight/obese), **(B)** diabetes duration (>10 years), and **(C)** medication adherence (low/no).

Pooled data from 11 studies showed significant odds of inadequate control among overweight/obese participants (OR: 1.17, 95% CI: 1.11–1.22, p < 0.001) with high heterogeneity (I^2^ = 68.80%, p <0.001) (**Figure 7**). Further, higher waist circumference was found to be significantly associated with inadequate control (OR: 1.09, 95% CI: 1.03–1.15, p < 0.001) with homogeneity (I^2^ = 0%, p = 0.72). Increased odds of inadequate glycaemic control were found among participants with longer diabetes duration (>10 years) (OR: 1.92, 95% CI: 1.68–2.16, p < 0.001), as well as in those receiving more than one OAD (OR: 2.13, 95% CI: 1.89–2.37, p < 0.001), OADs and insulin together (OR: 4.06, 95% CI: 2.58–5.54, p < 0.001), or insulin only (OR: 2.44, 95% CI: 1.70–3.19, p < 0.001) compared with those on a single OAD. Additionally, participants with low or no medication adherence (OR: 2.08, 95% CI: 1.61–2.54, p < 0.001), low dietary adherence (OR: 2.13, 95% CI: 1.33–2.93, p < 0.001), and physical inactivity (OR: 2.15, 95% CI: 1.35–2.95) had two-fold higher odds of inadequate glycaemic control compared with their counterparts (**Figure 7**). Furthermore, individuals with dyslipidaemia (OR: 1.43, 95% CI: 1.22–1.64, p < 0.001) or high low-density lipoprotein (LDL) levels (OR: 1.97, 95% CI: 0.97–2.96, p < 0.001) were also found to be at higher odds of inadequate glycaemic control. In all cases, the studies were found to be homogeneous (except for dyslipidaemia).

### 3.5 Subgroup analysis

**Supplementary Table 1** presents the subgroup analysis for inadequate glycaemic control by socio-demographic, behavioural, anthropometric and clinical variables. Participants with a history of smoking had a higher prevalence of inadequate glycaemic control (74%) compared with non-smokers (67%). Similarly, those with no or minimal physical activity had higher inadequate glycaemic control (73%) than those engaging in moderate/vigorous activity (63%). Diet and treatment adherence were also significant, with non-adherent individuals having higher inadequate glycaemic control (diet: 78% vs 63%; treatment: 76% vs 67%). Obese participants showed higher inadequate control (71%) compared with those with a normal body mass index (BMI) (63%), though the difference was not statistically significant. No publication bias was detected. Inadequate glycaemic control was higher in those with diabetes for ≥10 years (74%) versus those with a shorter duration (63%) and in participants treated with both OAD and insulin (81%) compared with those treated with a single OAD (59%). Participants with dyslipidaemia had higher inadequate glycaemic control than those without it. In contrast, participants without hypertension had higher inadequate glycaemic control than those with hypertension. In both cases, the differences were non-significant. Regarding diabetes-related complications, participants having retinopathy had the highest prevalence of inadequate control, followed by neuropathy, nephropathy and coronary artery disease respectively. Heterogeneity was high (>86%) across all subgroup analyses.

Furthermore, analysis was performed on the quality of the studies to identify possible sources of substantial/considerable heterogeneity. The pooled prevalence of inadequate glycaemic control was higher for studies ranked as good (70%, 95% CI: 63%–76%) compared with those ranked as fair (68%, 95% CI: 63%–72%) and poor (69%, 95% CI: 63%–76%). However, high heterogeneity was present (I^2^ > 99%) with minimal publication bias.

## 4 Discussion

This systematic review and meta-analysis examined the literature on glycaemic control and its related factors in people with T2DM in LMICs. The results showed that in most studies from LMICs, overall, more than two-thirds (69%) of people with diabetes had inadequate glycaemic control. By income level of the country, the pooled proportion of participants with inadequate glycaemic control was 68% in upper- middle-income countries, 69% in lower-middle-income countries, and 75% in low-income countries. Regionally, the proportion of participants with inadequate glycaemic control was highest in Africa (72%). In addition, inadequate glycaemic control was associated with sociodemographic factors (older age, female gender, education level below secondary and rural residence), lifestyle and behavioural factors (physical inactivity, dietary non-adherence, and medication non-adherence) and clinical factors (longer duration of diabetes, treatment modalities, high BMI and presence of dyslipidaemia).

This review highlights the significant challenge in controlling glycaemic levels among people with T2DM living in LMICs, with 69% failing to achieve the recommended HbA1c target. Previous review that included studies from sub-Saharan Africa also reported a similar pooled prevalence of people with inadequate control of 70% (10), and attributed this to the quality of diabetes care and fragmented health systems in these countries (82). Another systematic review conducted with studies in Ethiopia also reported similar prevalence (66.6%) and identified poor medication adherence, low education and health literacy as contributing factors (83). In line with these results, Manne-Goehler et al. (2019) reported that 77% of individuals with diabetes across 28 LMICs had inadequate glycaemic control (HbA1c ≥8%), based on nationally representative, community-based surveys (13). Compared to the current review, which largely includes facility-based studies, the higher rate in Manne-Goehler et al.’s study may reflect differences in study setting, sampling strategy, and access to care. Facility-based studies typically include individuals already diagnosed and linked to care, whereas community-based surveys capture a broader and more representative population—including those undiagnosed or not receiving regular care. Limited access to essential diagnostics, such as HbA1c testing, remains a major barrier to improving diabetes outcomes in these settings (84). This review also included studies that used various HbA1c thresholds (≥6.5%, ≥7%, or ≥8%) to define inadequate glycaemic control, further contributing to heterogeneity. Notable variations in prevalence were also observed across studies. For example, a hospital-based study in South Africa reported a high prevalence (83%)among participants with high obesity rates (BMI >30 kg/m²: 63% and central obesity: 95.4%) and complex treatment regimens combining insulin and metformin (56), whereas a study in Nigeria reported a lower prevalence (40%) among participants with lower obesity rates (18%), simpler treatment modalities, and better medication adherence (48). These contrasting findings underscore the influence of clinical characteristics, obesity burden, and treatment complexity on glycaemic outcomes, even within similar care settings.

The high heterogeneity observed across studies, despite multiple subgroup and sensitivity analyses, highlights the complex and diverse nature of diabetes management in LMICs. This variation likely reflects differences in healthcare infrastructure, diagnostic criteria, data collection methods, and population characteristics. Furthermore, one-third of the included studies were rated as poor quality, and nearly all were cross-sectional, limiting causal inference. Many studies also had small sample sizes and incomplete adjustment for potential confounders, which may have introduced bias and reduced the precision of effect estimates. Consequently, the pooled estimates and observed associations should be interpreted with caution, as study quality directly affects the reliability and generalisability of the findings. These methodological limitations underscore the need for future large-scale, longitudinal studies using robust designs and rigorous control for confounding factors.

The findings of this review underscore ongoing challenges in achieving glycaemic control in LMICs, particularly when compared to high-income countries (HICs), where control rates typically range from 52.2%–53.6% due to better health infrastructure, regular monitoring, access to newer therapies, and comprehensive patient education (7–9). In contrast, LMICs face systemic barriers such as financial constraints, understaffed health systems, and weak integration of diabetes care into primary health services, all of which contribute to suboptimal outcomes (7). However, data from the United States and European countries show that, even with advanced treatments and monitoring tools, a substantial proportion of people with diabetes still fail to meet the HbA1c target of <7% due to disease complexity, comorbidities, individual variability, and behavioural challenges such as poor adherence) (85). Therefore, this target may not be universally appropriate, particularly in low-resource settings. In populations with limited access to blood glucose monitoring and healthcare support, strict glycaemic targets may increase the risk of hypoglycaemia, particularly among those using sulfonylureas or insulin. Current guidelines emphasise individualising treatment goals based on patient characteristics, comorbidities, and healthcare system capacity (86). Thus, the threshold used in this review should be interpreted within the context of local resources and patient safety considerations.

In this review, 35 factors were identified as being associated with inadequate glycaemic control. The most commonly associated factors included longer diabetes duration, older age, higher BMI (overweight/obesity), female gender and various diabetes treatment modalities (OADs/OADs and insulin/insulin only). Additionally, low medication adherence and the presence of dyslipidaemia were notable contributors. Other factors, though less frequently reported, included low income, rural residence, below secondary education, high waist circumference, physical inactivity, dietary non-adherence and the presence of micro- and macrovascular complications. While individual-level factors were commonly assessed, important system-level and contextual factors—such as access to care, service continuity, quality and coordination of care, availability of healthcare providers (including community health workers), and person-centred service delivery—were not consistently measured across studies. These dimensions are particularly relevant in LMICs, where fragmented health systems and limited resources can substantially affect diabetes outcomes. Incorporating these healthcare delivery aspects into future research is essential to better understand the drivers of inadequate glycaemic control.

The current review found that older age was associated with inadequate glycaemic control, which could be explained by the natural decline in pancreatic β-cell function, increased prevalence of comorbidities, interference with diabetes management due to multiple medications, cognitive decline, reduced physical activity and dietary changes (14, 83). It is important to consider that glycaemic targets should be individualised, particularly in older adults, where strict control may increase the risk of hypoglycaemia. Current clinical guidelines recommend more relaxed glycaemic goals in this population, balancing the benefits of glucose lowering with potential treatment-related risks (86). Therefore, the interpretation of inadequate control should be contextualised within patient age, comorbidity burden, and functional status, rather than applying a uniform target across all subgroups. This review also found that women tended to have inadequate glycaemic control, which is consistent with the findings from previous research in Ethiopia (87). This could be due to hormonal changes throughout their lifespan and experience of higher levels of stress, depression and anxiety compared with men, which can affect insulin sensitivity and glucose metabolism (88). Additionally, some socioeconomic factors (e.g. lower income, limited access to healthcare and disparities in education) and lifestyle factors (e.g. poor dietary intake, less physical activity) may impact their ability to manage diabetes effectively (89, 90). Moreover, some women may prioritise the health of others before themselves, and their responsibilities such as caregiving and household duties may leave them with less time and energy to focus on self-care. Participants with education levels below secondary and those living in rural areas also had inadequate glycaemic control, which is supported by a previous study (24). Lower levels of education may impact health literacy and the ability to understand and adhere to treatment plans, as well as adopt healthy lifestyle behaviours (24). Limited healthcare facilities, shortages of healthcare professionals, transportation problems and longer travel distances in rural areas can result in suboptimal management and irregular follow-up (24). Regarding lifestyle and behavioural factors, inadequate control was associated with non-adherence to the recommended diet and prescribed medications. Poor medication adherence can be attributed to people-related factors, such as accessibility and affordability of medications, dosage and quantity of medicines and knowledge about their illness and medications. It can also be due to provider-related factors, such as the lack of continuous support and awareness regarding the disease and the importance of adherence to medication (38). These results highlight the need to identify people with T2DM who are socially disadvantaged, improve access to healthcare and implement effective self-care management strategies for people with varying health literacy.

Participants with a high BMI (overweight/obese) were more likely to have inadequate glycaemic control compared with those with a normal BMI, consistent with findings from previous studies (91). Obesity is a complex, multifactorial condition, and its association with inadequate glycaemic control in T2DM is mediated by several interrelated mechanisms. Excess adiposity contributes to insulin resistance through increased free fatty acid flux, ectopic fat deposition, and chronic low-grade inflammation driven by adipokines and pro-inflammatory cytokines such as tumour necrosis factor-alpha (TNF-α) and interleukin-6 (IL-6) (92–94). These processes impair glucose uptake in peripheral tissues and reduce insulin sensitivity, thereby making it more difficult to achieve optimal HbA1c levels even with pharmacological therapy. Additionally, obesity is often accompanied by increased hepatic fat accumulation, dyslipidaemia, physical inactivity, and excessive caloric intake, all of which further exacerbate hyperglycaemia and insulin resistance (95). Managing both obesity and T2DM often requires a comprehensive approach that includes weight management, physical activity, dietary changes and medication when necessary. Healthcare providers can help individuals in developing personalised management plans to address both conditions and improve glycaemic control.

Participants with longer duration of diabetes (>10 years) were found to have inadequate glycaemic control, which may be explained by the progressive nature of β-cell dysfunction and declining insulin secretion over time. Chronic exposure to hyperglycaemia and elevated free fatty acids leads to glucotoxicity, lipotoxicity, oxidative stress, and islet inflammation, all of which accelerate β-cell failure (96, 97). In addition, persistent insulin resistance, often exacerbated by aging and adiposity, further impairs glucose homeostasis (92). These progressive metabolic alterations collectively make it increasingly difficult to maintain optimal glycaemic control as the duration of diabetes lengthens. Furthermore, individuals with longer disease duration are more likely to develop comorbidities and complications that can interfere with diabetes self-management, medication adjustment, and overall metabolic control (98).

This review also found that participants on combinations of OAD and/or insulin had inadequate glycaemic control. The use of higher- intensity glucose-lowering medications may reflect the severity of the disease and the difficulty of achieving adequate glycaemic control while balancing possible risks of treatment, such as hypoglycaemia. A few of the more recent studies included in this review from countries such as Bangladesh (2021) (26), India (2021) (30), and Ethiopia (2019) (38) did report the use of newer classes of oral antidiabetic drugs (OADs), including sodium-glucose co-transporter-2 inhibitors (SGLT2i), dipeptidyl peptidase-4 inhibitors (DPP-4i), and glucagon-like peptide-1 receptor agonists (GLP-1RA). However, these reports were limited, and access to these newer agents remains constrained in many low- and middle-income country settings, potentially contributing to suboptimal treatment outcomes. Participants who had dyslipidaemia had also inadequate glycaemic control. Dyslipidaemia, particularly elevated levels of TG and LDL, is often associated with insulin resistance and poorer glycaemic control (99).

Interestingly, the study found that individuals without hypertension had a higher prevalence of inadequate glycaemic control compared to those with hypertension. This counterintuitive result may be explained by the increased frequency of healthcare interactions and more comprehensive disease management among patients with multiple comorbidities. Those with both diabetes and hypertension are more likely to be under routine monitoring, receive medication adjustments, and adhere to lifestyle changes, which may contribute to better glycaemic control. However, it is important to note that being under treatment does not necessarily translate into achieving target glycaemic levels, highlighting the need for ongoing evaluation of treatment effectiveness and patient adherence.

Despite differences in disease pathophysiology, comparisons with successful chronic disease programs—such as HIV viral suppression initiatives—highlight the urgent need for strengthened diabetes care systems in LMICs (100, 101). The consistently low glycaemic control rates observed across time and settings in this review underscore significant gaps in access, continuity, and quality of diabetes management. These findings point to the need for integrated, scalable, and patient-centred approaches—similar to those used in HIV care—that prioritise regular monitoring, adherence support, task-shifting to community health workers, and decentralised care delivery (102, 103). Future research should focus not only on individual-level predictors but also on evaluating health system interventions that can sustainably improve glycaemic outcomes in resource-constrained settings.

### 4.1 Strengths and limitations

This review has several limitations. First, while the review exclusively incorporated studies that measured glycaemic control using HbA1c, it is important to note that variations in the HbA1c test methods and cut-off values could potentially introduce errors in the estimates. Additionally, differences in HbA1c levels across ethnic and racial groups may affect comparability, as certain populations may exhibit varying HbA1c levels independent of glucose concentrations (104). Second, it is crucial to recognise that all the studies included in this review were cross-sectional (except one retrospective cohort survey), which limits the ability to establish causality between the identified factors and glycaemic control. The associations observed may reflect consequences rather than causes. For instance, individuals with high BMI or comorbidities such as dyslipidaemia may already have poor glycaemic outcomes, making it difficult to determine directionality. Similarly, treatment-related factors—such as the use of oral hypoglycaemic agents—may indicate poor control rather than predict it, introducing potential reverse causality. Third, the majority of the included studies were not community-based; only four were. Most studies used selected populations from hospitals or primary care centres, which may represent individuals already linked to care and thus fail to capture the broader population, particularly those who are undiagnosed or untreated. Limited access to diagnostics, such as HbA1c testing, in LMICs may further contribute to underestimating the true burden of poor glycaemic control. As a result, the generalisability of the findings to the wider community is limited. Fourth, significant heterogeneity was observed among some of the categories of studies, and despite conducting various subgroup analyses, the sources of heterogeneity could not be determined. This persistent heterogeneity likely reflects diversity in study populations, measurement tools, clinical practices, healthcare access, and unmeasured contextual factors across LMICs. Several potentially relevant variables—such as health system differences, quality of care, health insurance coverage, medication availability, and cultural influences—were not consistently reported, limiting further exploration. Fifth, the exclusion of grey literature such as theses, conference abstracts, and unpublished studies, may have introduced publication bias by omitting potentially relevant but non-peer-reviewed evidence. Finally, none of the studies addressed missing data or used newly emerged machine learning methods to identify the predictors of glycaemic control.

Despite these limitations, to the best of our knowledge, this systematic review and meta-analysis is the first comprehensive attempt to estimate the prevalence of inadequate glycaemic control and its associated factors in people with T2DM in LMICs. It should be noted that this review exclusively considered studies that conducted multivariable analyses during data analysis, thus excluding factors lacking a clear link to glycaemic control.

## 5 Conclusion

The overall prevalence of people with T2DM in LMICs with inadequate glycaemic control was 69%, underscoring a significant public health challenge. Poor glycaemic control is influenced by a range of sociodemographic, lifestyle, clinical and treatment-related factors. Addressing these determinants is the key to achieving better glycaemic control and reducing diabetes-related complications. However, service delivery factors—such as access, continuity, care coordination, and the role of community health systems—remain underexplored despite their potential impact. Future research should prioritise community-based studies that incorporate both individual and health system-level variables to more comprehensively understand the prevalence and factors associated with inadequate glycaemic control among disadvantaged populations within LMICs.

## Data Availability

The original contributions presented in the study are included in the article/**Supplementary Material**. Further inquiries can be directed to the corresponding author.

## Supplementary material

Supplementary Figure 1 Prevalence of inadequate glycaemic control (defined as HbA1c ≥6.5%, ≥7%, or ≥8%) by country.

Supplementary Figure 2 Prevalence of inadequate glycaemic control (defined as HbA1c ≥6.5%, ≥7%, or ≥8%) by study years.

Supplementary Figure 3 Pooled prevalence of inadequate glycaemic control (defined as HbA1c ≥6.5%, ≥7%, or ≥8%) stratified by study setting.
