# Supplementary material for: Glycaemic control and its related factors among people with type 2 diabetes in low- and middle-income countries: a systematic review and meta-analysis
Source: Front Clin Diabetes Healthc. 2025 Nov 27;6:1695235. doi: 10.3389/fcdhc.2025.1695235 (PMC12695616; doi:10.3389/fcdhc.2025.1695235)
Supplement: Supplementary Figure 1 — Prevalence of inadequate glycaemic control (defined as HbA1c ≥6.5%, ≥7%, or ≥8%) by country. [file DataSheet1.pdf]

Supplementary Material

1 Appendix 1: PSISMA checklist

Section and Topic	Item #	Checklist item	Location where item is reported
**TITLE**			
Title	1	Identify the report as a systematic review.	Page 1
**ABSTRACT**			
Abstract	2	See the PRISMA 2020 for Abstracts checklist.	Page 2
**INTRODUCTION**			
Rationale	3	Describe the rationale for the review in the context of existing knowledge.	Page 3-4
Objectives	4	Provide an explicit statement of the objective(s) or question(s) the review addresses.	Page 4
**METHODS**			
Eligibility criteria	5	Specify the inclusion and exclusion criteria for the review and how studies were grouped for the syntheses.	Page 4, 5
Information sources	6	Specify all databases, registers, websites, organisations, reference lists and other sources searched or consulted to identify studies. Specify the date when each source was last searched or consulted.	Page 5
Search strategy	7	Present the full search strategies for all databases, registers and websites, including any filters and limits used.	Page 5
Selection process	8	Specify the methods used to decide whether a study met the inclusion criteria of the review, including how many reviewers screened each record and each report retrieved, whether they worked independently, and if applicable, details of automation tools used in the process.	Page 5
Data collection process	9	Specify the methods used to collect data from reports, including how many reviewers collected data from each report, whether they worked independently, any processes for obtaining or confirming data from study investigators, and if applicable, details of automation tools used in the process.	Page 5
Data items	10a	List and define all outcomes for which data were sought. Specify whether all results that were compatible with each outcome domain in each study were sought (e.g. for all measures, time points, analyses), and if not, the methods used to decide which results to collect.	Page 5, 6
	10b	List and define all other variables for which data were sought (e.g. participant and intervention characteristics, funding sources). Describe any assumptions made about any missing or unclear information.	Page 5, 6
Study risk of bias assessment	11	Specify the methods used to assess risk of bias in the included studies, including details of the tool(s) used, how many reviewers assessed each study and whether they worked independently, and if applicable, details of automation tools used in the process.	Page 6

Section and Topic	Item #	Checklist item	Location where item is reported
Effect measures	12	Specify for each outcome the effect measure(s) (e.g. risk ratio, mean difference) used in the synthesis or presentation of results.	Page 6
Synthesis methods	13a	Describe the processes used to decide which studies were eligible for each synthesis (e.g. tabulating the study intervention characteristics and comparing against the planned groups for each synthesis (item #5)).	Page 6
	13b	Describe any methods required to prepare the data for presentation or synthesis, such as handling of missing summary statistics, or data conversions.	Page 6
	13c	Describe any methods used to tabulate or visually display results of individual studies and syntheses.	
	13d	Describe any methods used to synthesize results and provide a rationale for the choice(s). If meta-analysis was performed, describe the model(s), method(s) to identify the presence and extent of statistical heterogeneity, and software package(s) used.	Page 6
	13e	Describe any methods used to explore possible causes of heterogeneity among study results (e.g. subgroup analysis, meta-regression).	Page 6
	13f	Describe any sensitivity analyses conducted to assess robustness of the synthesized results.	Page 6
Reporting bias assessment	14	Describe any methods used to assess risk of bias due to missing results in a synthesis (arising from reporting biases).	
Certainty assessment	15	Describe any methods used to assess certainty (or confidence) in the body of evidence for an outcome.	
**RESULTS**			
Study selection	16a	Describe the results of the search and selection process, from the number of records identified in the search to the number of studies included in the review, ideally using a flow diagram.	Page 7
	16b	Cite studies that might appear to meet the inclusion criteria, but which were excluded, and explain why they were excluded.	
Study characteristics	17	Cite each included study and present its characteristics.	Table: Appendix 4
Risk of bias in studies	18	Present assessments of risk of bias for each included study.	
Results of individual studies	19	For all outcomes, present, for each study: (a) summary statistics for each group (where appropriate) and (b) an effect estimate and its precision (e.g. confidence/credible interval), ideally using structured tables or plots.	Figure 2
Results of syntheses	20a	For each synthesis, briefly summarise the characteristics and risk of bias among contributing studies.	Page 7-10
	20b	Present results of all statistical syntheses conducted. If meta-analysis was done, present for each the summary estimate and its precision (e.g. confidence/credible interval) and measures of statistical heterogeneity. If comparing groups, describe the direction of the effect.	Figure 2
	20c	Present results of all investigations of possible causes of heterogeneity among study results.	Page 10,

Section and Topic	Item #	Checklist item	Location where item is reported
			Supp table 1
	20d	Present results of all sensitivity analyses conducted to assess the robustness of the synthesized results.	Page 10
Reporting biases	21	Present assessments of risk of bias due to missing results (arising from reporting biases) for each synthesis assessed.	
Certainty of evidence	22	Present assessments of certainty (or confidence) in the body of evidence for each outcome assessed.	
**DISCUSSION**			
Discussion	23a	Provide a general interpretation of the results in the context of other evidence.	Page 10-15
	23b	Discuss any limitations of the evidence included in the review.	Page 15-16
	23c	Discuss any limitations of the review processes used.	Page 15-16
	23d	Discuss implications of the results for practice, policy, and future research.	Page 16
**OTHER INFORMATION**			
Registration and protocol	24a	Provide registration information for the review, including register name and registration number, or state that the review was not registered.	Page 4
	24b	Indicate where the review protocol can be accessed, or state that a protocol was not prepared.	N/A
	24c	Describe and explain any amendments to information provided at registration or in the protocol.	N/A
Support	25	Describe sources of financial or non-financial support for the review, and the role of the funders or sponsors in the review.	Page 17
Competing interests	26	Declare any competing interests of review authors.	Page 17
Availability of data, code and other materials	27	Report which of the following are publicly available and where they can be found: template data collection forms; data extracted from included studies; data used for all analyses; analytic code; any other materials used in the review.	Page 17

From: Page MJ, McKenzie JE, Bossuyt PM, Boutron I, Hoffmann TC, Mulrow CD, et al. The PRISMA 2020 statement: an updated guideline for reporting systematic reviews. BMJ 2021;372:n71. doi: 10.1136/bmj.n71

For more information, visit: http://www.prisma-statement.org/

Appendix 2: Search strategy for all five databases

#	**Search History (MEDLINE)**
1	Diabetes Mellitus, Type 2/
2	(diabet* adj1 (type 2 or type ii)).mp.
3	(T2DM or Tiidm or niddm).mp.
4	(noninsulin depend* or non-insulin depend* or mature-onset diabet* or adult-onset diabet*).mp.
5	1 OR 2 OR 3 OR 4
6	glyc?emic control/
7	(hb a1* or hba1* or h?emoglobinA1* or h?emoglobin A1*).mp.
8	(mean HbA1c*or mean absolute change in HbA1c or mean difference in HbA1c) [tiab]
9	(glycoh?emoglobin or glycated h?emoglobin or glycated h?emoglobin A or glycated h?emoglobinA) [tiab]
10	(glycemic targets or glycaemic targets[tiab]
11	(blood glucose levels or mean glucose level or blood sugar levels or blood glucose control [tiab]
12	6 OR 7 OR 8 OR 9 OR 10 OR 11
13	(resource-limit* or resource-poor or low-resource* or limited-resource* or resource-constrain* or constrain*-resource* or under-resource* or poor*-resource* or resource-scarce* or scarce*-resource* or low-income or middle-income or lowincome or middleincome or (low adj3 middle-income)).mp.
14	((developing or underdeveloped or under-developed or emerging or less-developed or least-developed or less-economically developed or least-economically developed or less-affluent or least-affluent or deprived or poor) adj (country or countries or nation or nations or region or regions or economy or economies)).mp.
15	((developing or underdeveloped or under-developed or less-developed or least-developed) adj (population* or world)).mp.
16	(third-world* or thirdworld* or 3rd-world* or lmic or lmics or lami countr* or lalmi countr* or transitional countr*).mp.
17	(low* adj (gdp or gnp or gross domestic or gross national)).mp. OR ((underserved or under served) adj (countr* or nation? or population*)).mp.
18	((developing or low* income or middle income) adj (countr* or nation?)).mp.
19	((developing or third) adj world).mp.
20	Afghan*[tw] OR Angola*[tw] OR Angolese*[tw] OR Angolian*[tw] OR Armenia*[tw] OR Bangladesh*[tw] OR Benin*[tw] OR Bhutan*[tw] OR Birma*[tw] OR Burma*[tw] OR Birmese*[tw] OR Burmese*[tw] OR Boliv*[tw] OR Botswan*[tw] OR burkina Faso*[tw] OR Burundi*[tw] OR Cabo Verde*[tw] OR Cambod*[tw] OR Cameroon*[tw] OR Cape Verd*[tw] OR Central Africa*[tw] OR Chad[tw] OR Comoro*[tw] OR Congo*[tw] OR Cote d'Ivoire*[tw] OR Djibouti*[tw] OR East Africa*[tw] OR Eastern Africa*[tw] OR Egypt*[tw] OR El Salvador*[tw] OR Equatorial Guinea*[tw] OR Eritre*[tw] OR Ethiopia*[tw] OR Gabon*[tw] OR Gambia*[tw] OR Gaza*[tw] OR "Georgia (Republic)"[Mesh] OR Ghan*[tw] OR Guatemal*[tw] OR Guinea[tw] OR Haiti*[tw] OR Hondur*[tw] OR India*[tw] OR Indones*[tw] OR Ivory Coast*[tw] OR Kenya*[tw] OR Kiribati*[tw] OR Kosovo*[tw] OR Kyrgyz*[tw] OR Lao PDR*[tw] OR Laos*[tw] OR Lesotho*[tw] OR Liberia*[tw] OR Madagascar*[tw] OR Malaw*[tw] OR Mali[tw] OR Mauritan*[tw] OR Mauriti*[tw] OR Micronesi*[tw] OR Mocambiqu*[tw] OR Moldov*[tw] OR Mongolia*[tw] OR Morocc*[tw] OR Mozambiqu*[tw] OR Myanmar*[tw] OR Namibia*[tw] OR Nepal*[tw] OR Nicaragua*[tw] OR Niger*[tw] OR North Korea*[tw] OR Northern Korea*[tw] OR (Democratic[tiab] AND People*[tiab] AND Republic of Korea[tiab]) OR "Democratic People's Republic of Korea"[Mesh] OR Pakistan*[tw] OR Papua New Guinea*[tw] OR Philippine*[tw] OR Principe[tw] OR Rhodesia*[tw] OR Rwanda*[tw] OR Samoa*[tw] OR Sao Tome*[tw] OR Senegal*[tw] OR Sierra Leone*[tw] OR Solomon Islands*[tw] OR Somalia*[tw] OR South Africa*[tw] OR South Sudan*[tw] OR Southern Africa*[tw] OR Sri Lanka*[tw] OR Sub Saharan Africa*[tw] OR Subsaharan Africa*[tw] OR Sudan*[tw] OR Swaziland*[tw] OR Syria*[tw] OR Tajikist*[tw] OR Tanzan*[tw] OR Timor*[tw] OR Togo*[tw] OR Tonga*[tw] OR Tunis*[tw] OR Ugand*[tw] OR Ukrain*[tw] OR Uzbekistan*[tw] OR Vanuatu*[tw] OR Vietnam*[tw] OR West Africa*[tw] OR West Bank*[tw] OR Western Africa*[tw] OR Yemen*[tw] OR Zaire*[tw] OR Zambia*[tw] OR Zimbabw*[tw])
21	13 OR 14 OR 15 OR 16 OR 17 OR 18 OR 19 OR 20
22	5 AND 12 AND 21
23	limit 22 to yr="2001 -Current"
24	limit 23 to "humans only (removes records about animals)"

Note: This search strategy was developed for MEDLINE

#	**Search History (Embase)**
1	non insulin dependent diabetes mellitus/
2	(diabet* adj1 (type 2 or type ii)).mp.
3	(T2DM or Tiidm or niddm).mp.
4	(noninsulin depend* or non-insulin depend* or mature-onset diabet* or adult-onset diabet*).mp.
5	1 OR 2 OR 3 OR 4
6	glyc?emic control/
7	(hb a1* or hba1* or h?emoglobinA1* or h?emoglobin A1*).mp.
8	(mean HbA1c*or mean absolute change in HbA1c or mean difference in HbA1c) [tiab]
9	(glycoh?emoglobin or glycated h?emoglobin or glycated h?emoglobin A or glycated h?emoglobinA) [tiab]
10	(glycemic targets or glycaemic targets[tiab]
11	(blood glucose levels or mean glucose level or blood sugar levels or blood glucose control [tiab]
12	6 OR 7 OR 8 OR 9 OR 10 OR 11

13	Developing Countries/
14	Afghan*[tw] OR Angola*[tw] OR Angolese*[tw] OR Angolian*[tw] OR Armenia*[tw] OR Bangladesh*[tw] OR Benin*[tw] OR Bhutan*[tw] OR Birma*[tw] OR Burma*[tw] OR Birmese*[tw] OR Burmese*[tw] OR Boliv*[tw] OR Botswan*[tw] OR burkina Faso*[tw] OR Burundi*[tw] OR Cabo Verde*[tw] OR Cambod*[tw] OR Cameroon*[tw] OR Cape Verd*[tw] OR Central Africa*[tw] OR Chad[tw] OR Comoro*[tw] OR Congo*[tw] OR Cote d'Ivoire*[tw] OR Djibouti*[tw] OR East Africa*[tw] OR Eastern Africa*[tw] OR Egypt*[tw] OR El Salvador*[tw] OR Equatorial Guinea*[tw] OR Eritre*[tw] OR Ethiopia*[tw] OR Gabon*[tw] OR Gambia*[tw] OR Gaza*[tw] OR "Georgia (Republic)"[Mesh] OR Ghan*[tw] OR Guatemal*[tw] OR Guinea[tw] OR Haiti*[tw] OR Hondur*[tw] OR India*[tw] OR Indones*[tw] OR Ivory Coast*[tw] OR Kenya*[tw] OR Kiribati*[tw] OR Kosovo*[tw] OR Kyrgyz*[tw] OR Lao PDR*[tw] OR Laos*[tw] OR Lesotho*[tw] OR Liberia*[tw] OR Madagascar*[tw] OR Malaw*[tw] OR Mali[tw] OR Mauritan*[tw] OR Mauriti*[tw] OR Micronesi*[tw] OR Mocambiqu*[tw] OR Moldov*[tw] OR Mongolia*[tw] OR Morocc*[tw] OR Mozambiqu*[tw] OR Myanmar*[tw] OR Namibia*[tw] OR Nepal*[tw] OR Nicaragua*[tw] OR Niger*[tw] OR North Korea*[tw] OR Northern Korea*[tw] OR (Democratic[tiab] AND People*[tiab] AND Republic of Korea[tiab]) OR "Democratic People's Republic of Korea"[Mesh] OR Pakistan*[tw] OR Papua New Guinea*[tw] OR Philippine*[tw] OR Principe[tw] OR Rhodesia*[tw] OR Rwanda*[tw] OR Samoa*[tw] OR Sao Tome*[tw] OR Senegal*[tw] OR Sierra Leone*[tw] OR Solomon Islands*[tw] OR Somalia*[tw] OR South Africa*[tw] OR South Sudan*[tw] OR Southern Africa*[tw] OR Sri Lanka*[tw] OR Sub Saharan Africa*[tw] OR Subsaharan Africa*[tw] OR Sudan*[tw] OR Swaziland*[tw] OR Syria*[tw] OR Tajikist*[tw] OR Tanzan*[tw] OR Timor*[tw] OR Togo*[tw] OR Tonga*[tw] OR Tunis*[tw] OR Ugand*[tw] OR Ukrain*[tw] OR Uzbekistan*[tw] OR Vanuatu*[tw] OR Vietnam*[tw] OR West Africa*[tw] OR West Bank*[tw] OR Western Africa*[tw] OR Yemen*[tw] OR Zaire*[tw] OR Zambia*[tw] OR Zimbabw*[tw])
15	((developing or low* income or middle income) adj (countr* or nation?)).mp.
16	((developing or third) adj world).mp.
17	(resource-limit* or resource-poor or low-resource* or limited-resource* or resource-constrain* or constrain*-resource* or under-resource* or poor*-resource* or resource-scarce* or scarce*-resource* or low-income or middle-income or lowincome or middleincome or (low adj3 middle-income)).mp.
18	((developing or underdeveloped or under-developed or emerging or less-developed or least-developed or less-economically developed or least-economically developed or less-affluent or least-affluent or deprived or poor) adj (country or countries or nation or nations or region or regions or economy or economies)).mp.
19	((developing or underdeveloped or under-developed or less-developed or least-developed) adj (population* or world)).mp.
20	(third-world* or thirdworld* or 3rd-world* or lmic or lmics or lami countr* or lalmi countr* or transitional countr*).mp.
21	(low* adj (gdp or gnp or gross domestic or gross national)).mp.
22	13 OR 14 OR 15 OR 16 OR 17 OR 18 OR 19 OR 20 OR 21
23	5 AND 12 AND 22
24	limit 23 to yr="2001 -Current"
25	limit 24 to "humans only (removes records about animals)"

Note: This search strategy was developed for Embase

#	**Search history (CINAHL)**
S19	Limiters - Publication Date: 20010101-20240415; Human
S18	S5 AND S8 AND S17
S17	S9 OR S10 OR S11 OR S12 OR S13 OR S14 OR S15
S16	(MH "Afghanistan") OR (MH "Angola" OR (MH "Armenia") OR (MH "Bangladesh") OR (MH "Benin") OR (MH "Bhutan") OR (MH "Burma") OR (MH "Bolivia") OR (MH "Botswana") OR (MH "Burkina Faso") OR (MH "Burundi") OR (MH "Cabo Verde") OR (MH "Cambodia") OR (MH "Cameroon") OR (MH "Cape Verde") OR (MH "Central Africa") OR (MH "Chad") OR (MH "Comoro") OR (MH "Congo") OR (MH "Cote d'Ivoire") OR (MH "Djibouti") OR (MH "East Africa") OR (MH "Eastern Africa") OR (MH "Egypt") OR (MH "El Salvador") OR (MH "Equatorial Guinea") OR (MH "Eritrea") OR (MH "Ethiopia") OR (MH "Gabon") OR (MH "Gambia") OR (MH "Gaza") OR (MH "Georgia") OR (MH "Ghana") OR (MH "Guatemala") OR (MH "Guinea") OR (MH "Haiti") OR (MH "Honduras") OR (MH "India") OR (MH "Indonesia") OR (MH "Ivory Coast") OR (MH "Kenya") OR (MH "Kiribati") OR (MH "Kosovo") OR (MH "Kyrgyz") OR (MH "Lao PDR") OR (MH "Laos") OR (MH "Lesotho") OR (MH "Liberia") OR (MH "Madagascar") OR (MH "Malawi") OR (MH "Mali") OR (MH "Mauritania") OR (MH "Mauritius") OR (MH "Micronesia") OR (MH "Moldova") OR (MH "Mongolia") OR (MH "Morocco") OR (MH "Mozambique") OR (MH "Myanmar") OR (MH "Namibia") OR (MH "Nepal") OR (MH "Nicaragua") OR (MH "Nigeria") OR (MH "North Korea") OR (MH "Democratic People's Republic of Korea"[Mesh]) OR (MH "Pakistan") OR (MH "Papua New Guinea") OR (MH "Philippines") OR (MH Principe") OR (MH "Rhodesia") OR (MH "Rwanda") OR (MH "Samoa") OR (MH "Sao Tome") OR (MH "Senegal") OR (MH "Sierra Leone") OR (MH "Solomon Islands") OR (MH "Somalia") OR (MH "South Africa") OR (MH "South Sudan") OR (MH "Sri Lanka") OR (MH "Sub Saharan Africa") OR (MH "Subsaharan Africa") OR (MH "Sudan") OR (MH "Swaziland") OR (MH "Syria") OR (MH "Tajikistan") OR (MH "Tanzania") OR (MH "Timor-Leste) OR (MH "Togo") OR (MH "Tonga") OR (MH "Tunisia") OR (MH "Uganda") OR (MH "Ukraine") OR (MH "Uzbekistan") OR (MH "Vanuatu") OR (MH "Vietnam") OR (MH "West Africa") OR (MH "West Bank") OR (MH "Yemen") OR (MH "Zaire") OR (MH "Zambia") OR (MH "Zimbabwe")
S15	((underserved or under served) N0 (countr* or nation? or population*))
S14	(low* N0 (gdp or gnp or gross domestic or gross national))
S13	(third-world* or thirdworld* or 3rd-world* or lmic or lmics or lami countr* or lalmi countr* or transitional countr*)
S12	((developing or underdeveloped or under-developed or less-developed or least-developed) N0 (population* or world))
S11	((developing or underdeveloped or under-developed or emerging or less-developed or least-developed or less-economically developed or least-economically developed or less-affluent or least-affluent or deprived or poor) N0 (country or countries or nation or nations or region or regions or economy or economies))

S10	("resource-limit*" or "resource-poor" or "low-resource*" or "limited-resource*" or "resource-constrain*" or "constrain*-resource*" or "under-resource*" or "poor*-resource*" or "resource-scarce*" or "scarce*-resource*" or "low-income" or "middle-income" or "lowincome" or "middleincome" or (low N3 "middle-income"))
S9	(MH "Developing Countries")
S8	S6 OR S7
S7	hba1c or glycated hemoglobin or hemoglobin a1c
S6	(MH "glycemic control") OR (MH "glycaemic control") OR (MH "blood sugar control") OR (MH "blood glucose control")
S5	S1 OR S2 OR S3 OR S4
S4	(“noninsulin depend*” or “non-insulin depend*” or “mature-onset diabet*” or “adult-onset diabet*”)
S3	(T2DM or Tiidm or niddm)
S2	(diabet* N1 (type 2 or type ii))
S1	(MH "Diabetes Mellitus, Type 2")

Note: This search strategy was developed for CINAHL

#	**Search History (PsychINFO)**
1	Diabetes Mellitus, Type 2/
2	(diabet* adj1 (type 2 or type ii)).mp.
3	(T2DM or Tiidm or niddm).mp.
4	(noninsulin depend* or non-insulin depend* or mature-onset diabet* or adult-onset diabet*).mp.
5	1 OR 2 OR 3 OR 4
6	glyc?emic control/
7	(hb a1* or hba1* or h?emoglobinA1* or h?emoglobin A1*).mp.
8	(mean HbA1c*or mean absolute change in HbA1c or mean difference in HbA1c) [tiab]
9	(glycoh?emoglobin or glycated h?emoglobin or glycated h?emoglobin A or glycated h?emoglobinA) [tiab]
10	(glycemic targets or glycaemic targets[tiab]
11	(blood glucose levels or mean glucose level or blood sugar levels or blood glucose control [tiab]
12	6 OR 7 OR 8 OR 9 OR 10 OR 11
13	(resource-limit* or resource-poor or low-resource* or limited-resource* or resource-constrain* or constrain*-resource* or under-resource* or poor*-resource* or resource-scarce* or scarce*-resource* or low-income or middle-income or lowincome or middleincome or (low adj3 middle-income)).mp.
14	((developing or underdeveloped or under-developed or emerging or less-developed or least-developed or less-economically developed or least-economically developed or less-affluent or least-affluent or deprived or poor) adj (country or countries or nation or nations or region or regions or economy or economies)).mp.
15	((developing or underdeveloped or under-developed or less-developed or least-developed) adj (population* or world)).mp.
16	(third-world* or thirdworld* or 3rd-world* or lmic or lmics or lami countr* or lalmi countr* or transitional countr*).mp.
17	(low* adj (gdp or gnp or gross domestic or gross national)).mp. OR ((underserved or under served) adj (countr* or nation? or population*)).mp.
18	((developing or low* income or middle income) adj (countr* or nation?)).mp.
19	((developing or third) adj world).mp.
20	Afghan*[tw] OR Angola*[tw] OR Angolese*[tw] OR Angolian*[tw] OR Armenia*[tw] OR Bangladesh*[tw] OR Benin*[tw] OR Bhutan*[tw] OR Birma*[tw] OR Burma*[tw] OR Birmese*[tw] OR Burmese*[tw] OR Boliv*[tw] OR Botswan*[tw] OR burkina Faso*[tw] OR Burundi*[tw] OR Cabo Verde*[tw] OR Cambod*[tw] OR Cameroon*[tw] OR Cape Verd*[tw] OR Central Africa*[tw] OR Chad[tw] OR Comoro*[tw] OR Congo*[tw] OR Cote d'Ivoire*[tw] OR Djibouti*[tw] OR East Africa*[tw] OR Eastern Africa*[tw] OR Egypt*[tw] OR El Salvador*[tw] OR Equatorial Guinea*[tw] OR Eritre*[tw] OR Ethiopia*[tw] OR Gabon*[tw] OR Gambia*[tw] OR Gaza*[tw] OR "Georgia (Republic)"[Mesh] OR Ghan*[tw] OR Guatemal*[tw] OR Guinea[tw] OR Haiti*[tw] OR Hondur*[tw] OR India*[tw] OR Indones*[tw] OR Ivory Coast*[tw] OR Kenya*[tw] OR Kiribati*[tw] OR Kosovo*[tw] OR Kyrgyz*[tw] OR Lao PDR*[tw] OR Laos*[tw] OR Lesotho*[tw] OR Liberia*[tw] OR Madagascar*[tw] OR Malaw*[tw] OR Mali[tw] OR Mauritan*[tw] OR Mauriti*[tw] OR Micronesi*[tw] OR Mocambiqu*[tw] OR Moldov*[tw] OR Mongolia*[tw] OR Morocc*[tw] OR Mozambiqu*[tw] OR Myanmar*[tw] OR Namibia*[tw] OR Nepal*[tw] OR Nicaragua*[tw] OR Niger*[tw] OR North Korea*[tw] OR Northern Korea*[tw] OR (Democratic[tiab] AND People*[tiab] AND Republic of Korea[tiab]) OR "Democratic People's Republic of Korea"[Mesh] OR Pakistan*[tw] OR Papua New Guinea*[tw] OR Philippine*[tw] OR Principe[tw] OR Rhodesia*[tw] OR Rwanda*[tw] OR Samoa*[tw] OR Sao Tome*[tw] OR Senegal*[tw] OR Sierra Leone*[tw] OR Solomon Islands*[tw] OR Somalia*[tw] OR South Africa*[tw] OR South Sudan*[tw] OR Southern Africa*[tw] OR Sri Lanka*[tw] OR Sub Saharan Africa*[tw] OR Subsaharan Africa*[tw] OR Sudan*[tw] OR Swaziland*[tw] OR Syria*[tw] OR Tajikist*[tw] OR Tanzan*[tw] OR Timor*[tw] OR Togo*[tw] OR Tonga*[tw] OR Tunis*[tw] OR Ugand*[tw] OR Ukrain*[tw] OR Uzbekistan*[tw] OR Vanuatu*[tw] OR Vietnam*[tw] OR West Africa*[tw] OR West Bank*[tw] OR Western Africa*[tw] OR Yemen*[tw] OR Zaire*[tw] OR Zambia*[tw] OR Zimbabw*[tw])
21	13 OR 14 OR 15 OR 16 OR 17 OR 18 OR 19 OR 20
22	5 AND 12 AND 21
23	limit 22 to yr="2001 -Current"
24	limit 23 to "humans only (removes records about animals)"

Note: This search strategy was developed for PsychINFO

#	**Search history (Global health)**
1	Diabetes Mellitus, Type 2/
2	(diabet* adj1 (type 2 or type ii)).mp.
3	(T2DM or Tiidm or niddm).mp.
4	(noninsulin depend* or non-insulin depend* or mature-onset diabet* or adult-onset diabet*).mp.
5	1 or 2 or 3 or 4
6	glyc?emic control/
7	(hb a1* or hba1* or h?emoglobinA1* or h?emoglobin A1*).mp.
8	(mean HbA1c*or mean absolute change in HbA1c or mean difference in HbA1c) [tiab]
9	(glycoh?emoglobin or glycated h?emoglobin or glycated h?emoglobin A or glycated h?emoglobinA) [tiab]
10	(glycemic targets or glycaemic targets[tiab]
11	(blood glucose levels or mean glucose level or blood sugar levels or blood glucose control [tiab]
12	6 or 7 or 8 or 9 or 10 or 11
13	Developing Countries/
14	((developing or low* income or middle income) adj (countr* or nation?)).mp.
15	((developing or third) adj world).mp.
16	(resource-limit* or resource-poor or low-resource* or limited-resource* or resource-constrain* or constrain*-resource* or under-resource* or poor*-resource* or resource-scarce* or scarce*-resource* or low-income or middle-income or lowincome or middleincome or (low adj3 middle-income)).mp.
17	((developing or underdeveloped or under-developed or emerging or less-developed or least-developed or less-economically developed or least-economically developed or less-affluent or least-affluent or deprived or poor) adj (country or countries or nation or nations or region or regions or economy or economies)).mp.
18	((developing or underdeveloped or under-developed or less-developed or least-developed) adj (population* or world)).mp.
19	(third-world* or thirdworld* or 3rd-world* or lmic or lmics or lami countr* or lalmi countr* or transitional countr*).mp.
20	(low* adj (gdp or gnp or gross domestic or gross national)).mp.
21	((underserved or under served) adj (countr* or nation? or population*)).mp.
22	Afghan*[tw] OR Angola*[tw] OR Angolese*[tw] OR Angolian*[tw] OR Armenia*[tw] OR Bangladesh*[tw] OR Benin*[tw] OR Bhutan*[tw] OR Birma*[tw] OR Burma*[tw] OR Birmese*[tw] OR Burmese*[tw] OR Boliv*[tw] OR Botswan*[tw] OR burkina Faso*[tw] OR Burundi*[tw] OR Cabo Verde*[tw] OR Cambod*[tw] OR Cameroon*[tw] OR Cape Verd*[tw] OR Central Africa*[tw] OR Chad[tw] OR Comoro*[tw] OR Congo*[tw] OR Cote d'Ivoire*[tw] OR Djibouti*[tw] OR East Africa*[tw] OR Eastern Africa*[tw] OR Egypt*[tw] OR El Salvador*[tw] OR Equatorial Guinea*[tw] OR Eritre*[tw] OR Ethiopia*[tw] OR Gabon*[tw] OR Gambia*[tw] OR Gaza*[tw] OR "Georgia (Republic)"[Mesh] OR Ghan*[tw] OR Guatemal*[tw] OR Guinea[tw] OR Haiti*[tw] OR Hondur*[tw] OR India*[tw] OR Indones*[tw] OR Ivory Coast*[tw] OR Kenya*[tw] OR Kiribati*[tw] OR Kosovo*[tw] OR Kyrgyz*[tw] OR Lao PDR*[tw] OR Laos*[tw] OR Lesotho*[tw] OR Liberia*[tw] OR Madagascar*[tw] OR Malaw*[tw] OR Mali[tw] OR Mauritan*[tw] OR Mauriti*[tw] OR Micronesi*[tw] OR Mocambiqu*[tw] OR Moldov*[tw] OR Mongolia*[tw] OR Morocc*[tw] OR Mozambiqu*[tw] OR Myanmar*[tw] OR Namibia*[tw] OR Nepal*[tw] OR Nicaragua*[tw] OR Niger*[tw] OR North Korea*[tw] OR Northern Korea*[tw] OR (Democratic[tiab] AND People*[tiab] AND Republic of Korea[tiab]) OR "Democratic People's Republic of Korea"[Mesh] OR Pakistan*[tw] OR Papua New Guinea*[tw] OR Philippine*[tw] OR Principe[tw] OR Rhodesia*[tw] OR Rwanda*[tw] OR Samoa*[tw] OR Sao Tome*[tw] OR Senegal*[tw] OR Sierra Leone*[tw] OR Solomon Islands*[tw] OR Somalia*[tw] OR South Africa*[tw] OR South Sudan*[tw] OR Southern Africa*[tw] OR Sri Lanka*[tw] OR Sub Saharan Africa*[tw] OR Subsaharan Africa*[tw] OR Sudan*[tw] OR Swaziland*[tw] OR Syria*[tw] OR Tajikist*[tw] OR Tanzan*[tw] OR Timor*[tw] OR Togo*[tw] OR Tonga*[tw] OR Tunis*[tw] OR Ugand*[tw] OR Ukrain*[tw] OR Uzbekistan*[tw] OR Vanuatu*[tw] OR Vietnam*[tw] OR West Africa*[tw] OR West Bank*[tw] OR Western Africa*[tw] OR Yemen*[tw] OR Zaire*[tw] OR Zambia*[tw] OR Zimbabw*[tw]

Note: This search strategy was developed for Global health

Appendix 3: Tool used for quality assessment

NEWCASTLE - OTTAWA QUALITY ASSESSMENT SCALE
(adapted for cross sectional studies)
Selection: (Maximum 3 stars)

1) Representativeness of the sample:

    a) Truly representative of the average in the target population (all subjects or random sampling). 1 star

    b) Somewhat representative of the average in the target population (non-random sampling). 1 star

    c) Selected group of users. 0 star

    d) No description of the sampling strategy. 0 star

2) Non-respondents:

a) Comparability between respondents and non-respondents characteristics is established, and the response rate is satisfactory. 1 star

b) The response rate is unsatisfactory, or the comparability between respondents and non-respondents is unsatisfactory.

c) No description of the response rate or the characteristics of the responders and the non-responders.

3) Ascertainment of the exposure (risk factor):

a) Validated measurement tool. 1 star

b) Non-validated measurement tool, but the tool is available or described. 0 star

c) No description of the measurement tool. 0 star

Comparability: (Maximum 2 stars)

1) The subjects in different outcome groups are comparable, based on the study design or analysis. Confounding factors are controlled.

a) The study controls for the most important factor (select one) confounders. 1 star

b) The study controls for any additional factor. does not investigate potential confounders. 1 star

Outcome: (Maximum 2 stars)

1) Assessment of the outcome:

a) Independent blind assessment. 1 scores

b) Record linkage. 1 scores

c) Self report. 0 score

d) No description. 0 score

2) Statistical test:

a) The statistical test used to analyse the data is clearly described and appropriate, and the measurement of the association is presented, including confidence intervals and the probability level (p value). 1 star

b) The statistical test is not appropriate, not described or incomplete. 0 star

***NEWCASTLE - OTTAWA QUALITY ASSESSMENT SCALE for cohort study was used for cohort survey**

Appendix 4: Quality rating for studies (cross-sectional) included in the review

Study	Selection criteria (maximum score=3)			Comparability (maximum score=2)	Outcome (maximum score=2)		Total* (7)
	Representativeness of the sample*	Non-respondent*	Ascertainment of the exposure*	The subjects in different outcome groups are comparable, based on the study design or analysis. Confounding factors are controlled.	Assessment of the outcome*	Statistical tests*	
Abd-Elraouf MSE, 2020	1	1	1	0	1	0	4
Abdullah NA et al, 2019	1	1	1	1	1	1	6
Abera RG et al, 2022	1	1	1	2	1	1	7
Adeniyi OV et al, 2016	1	1	1	2	1	1	7
Afroz A et al, 2019	1	1	1	2	1	1	7
Ahmad NS et al, 2014	1	1	1	2	1	1	7
Akter N, 2020	1	1	1	0	1	0	4
Al-Zurfi et al, 2012	1	0	0	1	1	1	4
Amsah et al, 2022	1	1	1	1	1	1	6
Artha et al, 2019	1	0	0	1	1	1	4
Ashraf et al, 2020	1	1	1	1	1	1	6
Ashur ST et al	1	1	1	2	1	1	7
Babaniamansour S et al, 2020	1	1	1	1	1	0	5
BeLue et al	1	0	0	1	1	1	4
Bi Y et al, 2010	1	1	1	0	1	0	4
Borgharkar SS and Das SS, 2019	1	1	1	2	1	1	7
Camara et al, 2014	1	1	1	2	1	1	7
Chetoui A et al, 2022	1	1	1	1	1	1	6
Chua SS and Chan SP, 2011	1	0	0	1	1	1	4
Demoz GT et al, 2019	1	1	0	2	1	1	6

**Study**	**Selection criteria (maximum score=3)**			**Comparability (maximum score=2)**	**Outcome (maximum score=2)**		**Total* (7)**
	Representativeness of the sample*	Non-respondent*	Ascertainment of the exposure*	The subjects in different outcome groups are comparable, based on the study design or analysis. Confounding factors are controlled.	Assessment of the outcome*	Statistical tests*	
Diaf M and Khaled BM, 2017	1	0	0	2	1	1	5
Djonor SK et al, 2021	1	1	1	2	1	1	7
Eid M et al, 2003	1	0	0	1	1	0	3
Firouzi S et al, 2015	1	0	0	1	1	1	4
Goyal J et al, 2019	1	0	0	1	1	1	4
Gumilas NSA et al, 2021	1	0	1	0	1	1	4
Gurjar SS et al, 2023	1	1	1	0	1	0	4
Hassan MR et al, 2021	1	0	0	1	1	1	4
Howteerakul N et al, 2007	1	1	1	2	1	1	7
Ibrahim et al, 2021	1	1	1	1	1	1	6
Ismail A et al, 2016	1	0	0	1	1	1	4
Khattab M et al, 2008	1	1	1	1	1	1	6
Kumar SP and Sandhya AM, 2017	1	0	1	0	1	1	4
Li J et al, 2018	1	1	1	2	1	1	7
Lima RF et al, 2016	1	0	1	2	1	1	6
Mashele TS et al, 2019	1	1	1	1	1	1	6
Mahmood et al, 2016	1	1	0	1	1	1	5
Maiftrianti et al, 2020	1	0	0	2	1	1	5
Mendes ABV et al, 2009	1	1	1	0	1	0	4
MH Nini Shuhaida et al, 2019	1	1	1	1	1	1	6
Mobula LM et al, 2018	1	0	1	1	1	1	5
Moreira Jr. et al, 2010	1	1	1	0	1	0	4

Study	Selection criteria (maximum score=3)			Comparability (maximum score=2)	Outcome (maximum score=2)		Total* (7)
	Representativeness of the sample*	Non-respondent*	Ascertainment of the exposure*	The subjects in different outcome groups are comparable, based on the study design or analysis. Confounding factors are controlled.	Assessment of the outcome*	Statistical tests*	
Mwavua SM et al, 2016	1	1	0	1	1	1	5
Najeeb SS et al, 2022	1	1	1	2	1	1	7
Nyunt SW et al, 2010	1	1	0	2	1	1	6
Noor SK et al, 2017	1	0	1	1	1	1	5
Omar SM et al, 2018	1	1	1	1	1	1	6
Patrick NB et al, 2021	1	1	1	2	1	1	7
Rossaneis MA et al, 2017	1	0	1	1	1	1	5
Saghir et al, 2019	1	1	1	1	1	1	6
Siddiqui FJ et al, 2014	1	1	1	2	1	1	7
Soffian et al, 2019	1	1	1	2	1	1	7
Tharek Z et al, 2018	1	1	1	2	1	1	7
Thaneerat T et al, 2009	1	1	1	1	1	1	6
Thuita AW, 2019	1	1	0	1	1	1	5
Ufuoma C et al, 2016	1	1	1	0	1	0	4
Viana LV et al, 2013	1	1	0	1	1	1	5
Wang J et al, 2021	1	0	0	2	1	1	5
WH WF et al, 2016	1	1	1	0	1	0	4
Xing et al, 2022	1	0	1	2	1	1	6
Yeemard F et al, 2022	1	1	1	2	1	1	7

*Cut-off values of 0-4, 5-6 and ≥7 were used to classify the studies as poor, fair and good quality, respectively.

Appendix 5: Quality rating for study (cohort) included in the review

**Study**	**Selection (maximum score=4)**				**Comparability (maximum score=2)**	**Outcome (maximum score=3)**			**Total (9)**
	Representativeness of the exposed cohort	Selection of the non exposed cohort	Ascertainment of the exposure*	Demonstration that outcome of interest was not present at start of study		Assessment of outcome	Was follow-up long enough for outcomes to occur	Adequacy of follow-up of cohorts	
Thuy LQ et al, 2021	1	1	1	1	0	1	1	1	7

*Cut-off values of 0-4, 5-6 and ≥7 were used to classify the studies as poor, fair and good quality, respectively.

2 Supplementary Figures and Table

2.1 Supplementary Table

Appendix 6: Factors associated with inadequate glycaemic control in the included studies

**Variables**	**First author, publication year**																	
	Abd-Elraouf, 2020	Abdullah, 2019	Abera, 2022	Adeniyi, 2016	Afroz, 2019	Ahmad, 2014	Akter, 2020	Al-Zurfi, 2012	Amsah, 2022	Artha, 2019	Ashraf, 2020	Ashur, 2016	Babaniamansour, 2020	BeLue, 2016	Bi Y, 2010	Borgharkar, 2019	Camara, 2014	Chetoui, 2022
**Socio-demographics**																		
Age			+				+		+					+	+		+	
Gender				+	+							+		+				
Education					+										+			
Occupation																		
Residence					+													

**Variables**	**First author, publication year**																	
	Abd-Elraouf, 2020	Abdullah, 2019	Abera, 2022	Adeniyi, 2016	Afroz, 2019	Ahmad, 2014	Akter, 2020	Al-Zurfi, 2012	Amsah, 2022	Artha, 2019	Ashraf, 2020	Ashur, 2016	Babaniamansour, 2020	BeLue, 2016	Bi Y, 2010	Borgharkar, 2019	Camara, 2014	Chetoui, 2022
Income				+											+			
Marital status																		
Ethnicity									+									
**Anthropometrics**																		
BMI	+			+				+	+						+	+		
WC																		
**Behavioural**																		
Smoking																		
Physical activity				+								+						
**Clinical**																		
Diabetes duration			+	+		+			+		+			+	+	+	+	+
Diabetes treatment																		
OADs						+	+								+		+	
OAD + insulin												+					+	+
Insulin only			+		+							+					+	+
Diabetes complications																		
Nephropathy											+							
Retinopathy											+				+			
Neuropathy											+				+			
CAD											+							
Stroke																		
Diabetic foot																		
Hypertension														+		+		
Dyslipidaemia									+		+							
**Biochemical**																		
FBS	+																	
TC	+									+								
TG										+								
LDL	+			+						+								
HDL										+								
eGFR																		
**Others**																		
SMBG															+			
Diabetes education															+			

**Variables**	**First author, publication year**																	
	Abd-Elraouf, 2020	Abdullah, 2019	Abera, 2022	Adeniyi, 2016	Afroz, 2019	Ahmad, 2014	Akter, 2020	Al-Zurfi, 2012	Amsah, 2022	Artha, 2019	Ashraf, 2020	Ashur, 2016	Babaniamansour, 2020	BeLue, 2016	Bi Y, 2010	Borgharkar, 2019	Camara, 2014	Chetoui, 2022
Medication adherence												+						
Dietary adherence			+		+													
Social support																		
Self-perception																		

Appendix 6: (continued)

**Variables**	**First author, publication year**																	
	Chua, 2011	Demoz, 2019	Diaf, 2017	Djonor, 2021	Eid, 2003	Firouzi, 2015	Goyal, 2019	Gumilas, 2021	Gurjar, 2023	Hassan, 2021	Howteerakul, 2007	Ibrahim, 2021	Ismail, 2016	Khattab, 2008	Kumar, 2017	Li, 218	Lima, 2016	Mashele, 2019
**Socio-demographics**																		
Age					+					+		+						
Gender		+								+								
Education													+					
Occupation																		
Residence																		
Income												+						
Marital status																		
Ethnicity																		
**Anthropometrics**																		
BMI		+										+						
WC			+															+
**Behavioural**																		
Smoking																		
Physical activity																		
**Clinical**																		
Diabetes duration	+				+													
Diabetes treatment																		
OADs			+			+						+						
OAD + insulin	+												+					
Insulin only	+											+						

Variables	First author, publication year																	
	Chua, 2011	Demoz, 2019	Diaf, 2017	Djonor, 2021	Eid, 2003	Firouzi, 2015	Goyal, 2019	Gumilas, 2021	Gurjar, 2023	Hassan, 2021	Howteera kul, 2007	Ibrahim, 2021	Ismail, 2016	Khattab, 2008	Kumar, 2017	Li, 218	Lima, 2016	Mas hele, 2019
Diabetes complications																		
Nephropathy																		
Retinopathy																		
Neuropathy																		
CAD																		
Stroke																		
Diabetic foot																		
Hypertension																		
Dyslipidaemia																		
**Biochemical**																		
FBS																		
TC																		
TG										+								
LDL																		
HDL						+				+								
eGFR																		
**Others**																		
SMBG																		
Diabetes education																		
Medication adherence	+	+										+						
Dietary adherence																		
Social support																		
Self-perception																		

Appendix 6: (continued)

Variables	First author, publication year																	
	Chua, 2011	Demoz, 2019	Diaf, 2017	Djonor, 2021	Eid, 2003	Firouzi, 2015	Goyal, 2019	Gumilas, 2021	Gurjar, 2023	Hassan, 2021	Howteera kul, 2007	Ibrahim, 2021	Ismail, 2016	Khattab, 2008	Kumar, 2017	Li, 218	Lima, 2016	Mas hele, 2019
**Socio-demographics**																		
Age					+					+		+						
Gender		+								+								
Education													+					

Occupation																		
Residence																		
Income												+						
Marital status																		
Ethnicity																		
**Anthropometrics**																		
BMI		+										+						
WC			+															+
**Behavioural**																		
Smoking																		
Physical activity																		
**Clinical**																		
Diabetes duration	+				+													
Diabetes treatment																		
OADs			+			+						+						
OAD + insulin	+												+					
Insulin only	+											+						
Diabetes complications																		
Nephropathy																		
Retinopathy																		
Neuropathy																		
CAD																		
Stroke																		
Diabetic foot																		
Hypertension																		
Dyslipidaemia																		
**Biochemical**																		
FBS																		
TC																		
TG										+								
LDL																		
HDL						+				+								
eGFR																		
**Others**																		
SMBG																		
Diabetes education																		
Medication adherence	+	+										+						

Dietary adherence																		
Social support																		
Self-perception																		

Appendix 6: (continued)

**Variables**	**First author, publication year**																	
	Mahmood, 2016	Maiftrianti, 2020	Mendes, 2019	MH Nini, 2019	Mobula, 2018	Moreira, 2010	Mwavua, 2016	Najeeb, 2022	Nyunt, 2010	Noor, 2017	Omar, 2018	Patrick, 2021	Rossaneis, 2017	Saghir, 2019	Siddiqui, 2014	Soffian, 2019	Tharek, 2018	Thaneerat, 2009
**Socio-demographics**																		
Age	+								+			+	+	+	+	+		
Gender	+							+						+		+		
Education														+				
Occupation				+														
Residence																		
Income																		
Marital status											+							
Ethnicity																+		
**Anthropometrics**																		
BMI	+								+				+					
WC																	+	
**Behavioural**																		
Smoking																		
Physical activity														+				
**Clinical**																		
Diabetes duration	+		+									+		+		+	+	
Diabetes treatment	+																	
OADs		+							+									
OAD + insulin								+				+						
Insulin only													+	+				
Diabetes complications																		
Nephropathy														+				+
Retinopathy																		+
Neuropathy																		
CAD																		
Stroke																		
Diabetic foot													+					

Variables	First author, publication year																	
	Mahmood, 2016	Maiftrianti, 2020	Mendes, 2019	MH Nini, 2019	Mobula, 2018	Moreira, 2010	Mwavua, 2016	Najeeb, 2022	Nyunt, 2010	Noor, 2017	Omar, 2018	Patrick, 2021	Rossaneis, 2017	Saghir, 2019	Siddiqui, 2014	Soffian, 2019	Tharek, 2018	Thaneerat, 2009
Hypertension																		+
Dyslipidaemia																		
**Biochemical**																		
FBS																		
TC	+										+							
TG	+																	
LDL	+																	+
HDL	+																	
eGFR																		
**Others**										+								
SMBG														+				
Diabetes education			+															
Medication adherence							+	+						+				
Dietary adherence														+				
Social support				+														
Self-perception						+			+								+	

Appendix 6: (continued)

Variables	First author, publication year							
	Thuita, 2019	Thuy, 2021	Ufuoma, 2016	Viana, 2013	Wang, 2021	WH WF, 2016	Xing, 2022	Yeemard, 2022
**Socio-demographics**								
Age				+				+
Gender								
Education								
Occupation								
Residence								
Income								
Marital status								
Ethnicity								
**Anthropometrics**								
BMI		+						

WC								
**Behavioural**								
Smoking								
Physical activity								
**Clinical**								
Diabetes duration			+	+				+
Diabetes treatment								
OADs								
OAD + insulin								
Insulin only				+				
Diabetes complications								
Nephropathy								
Retinopathy								
Neuropathy								
CAD								
Stroke								
Diabetic foot								
Hypertension	+							
Dyslipidaemia	+							
**Biochemical**								
FBS	+	+						
TC								
TG								
LDL								
HDL								
eGFR								
**Others**								
SMBG				+				
Diabetes education			+					
Medication adherence							+	+
Dietary adherence								
Social support								
Self-perception						+		

2.2 Supplementary Figures

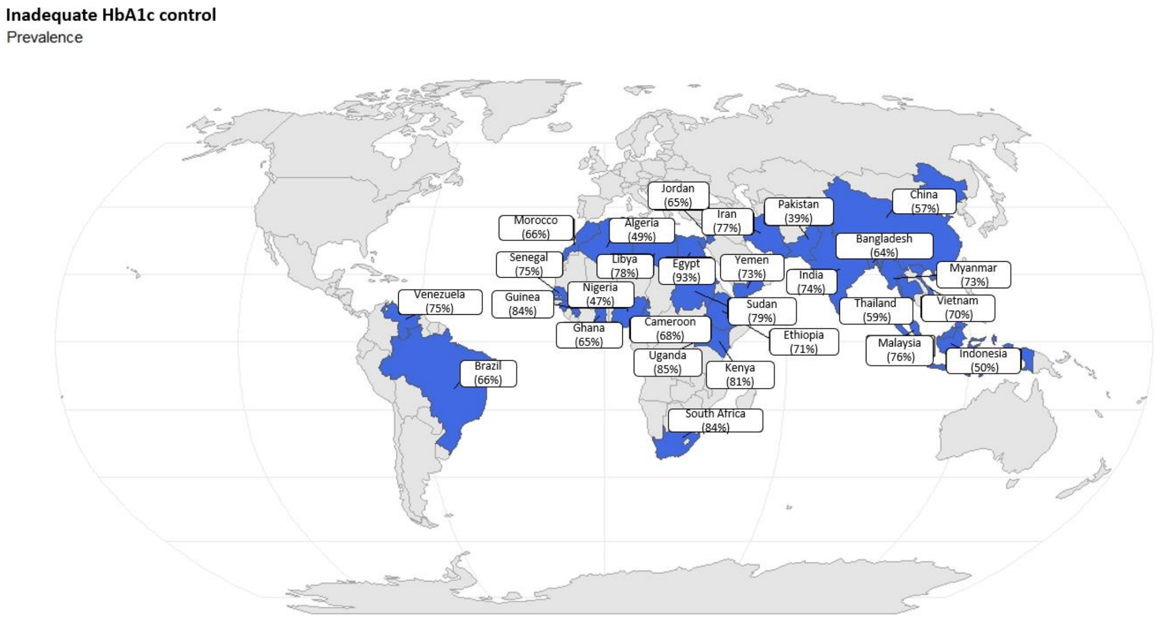

Supplementary figure 1: Prevalence of inadequate glycaemic control (defined as HbA1c ≥6.5%, ≥7%, or ≥8%) by country

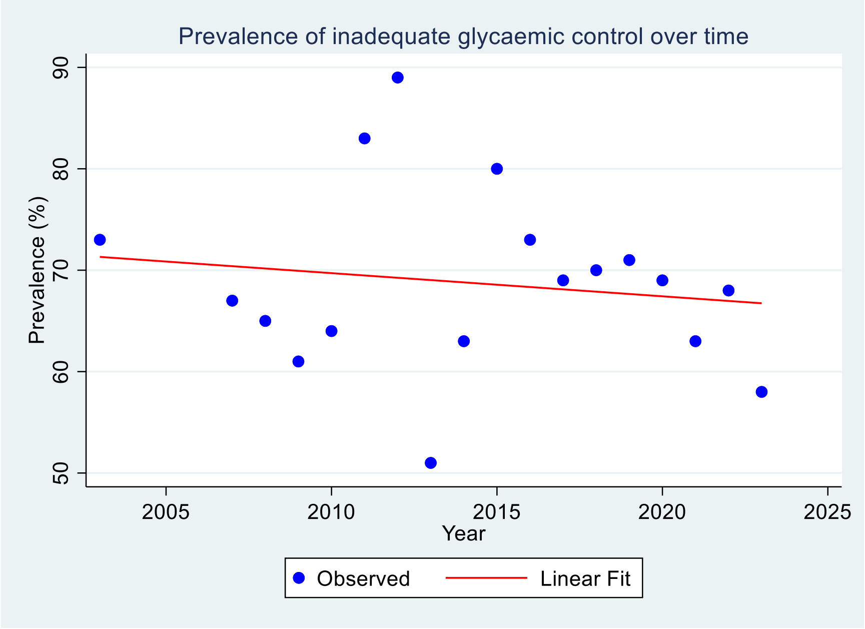

Supplementary figure 2: Prevalence of inadequate glycaemic control (defined as HbA1c ≥6.5%, ≥7%, or ≥8%) by study years

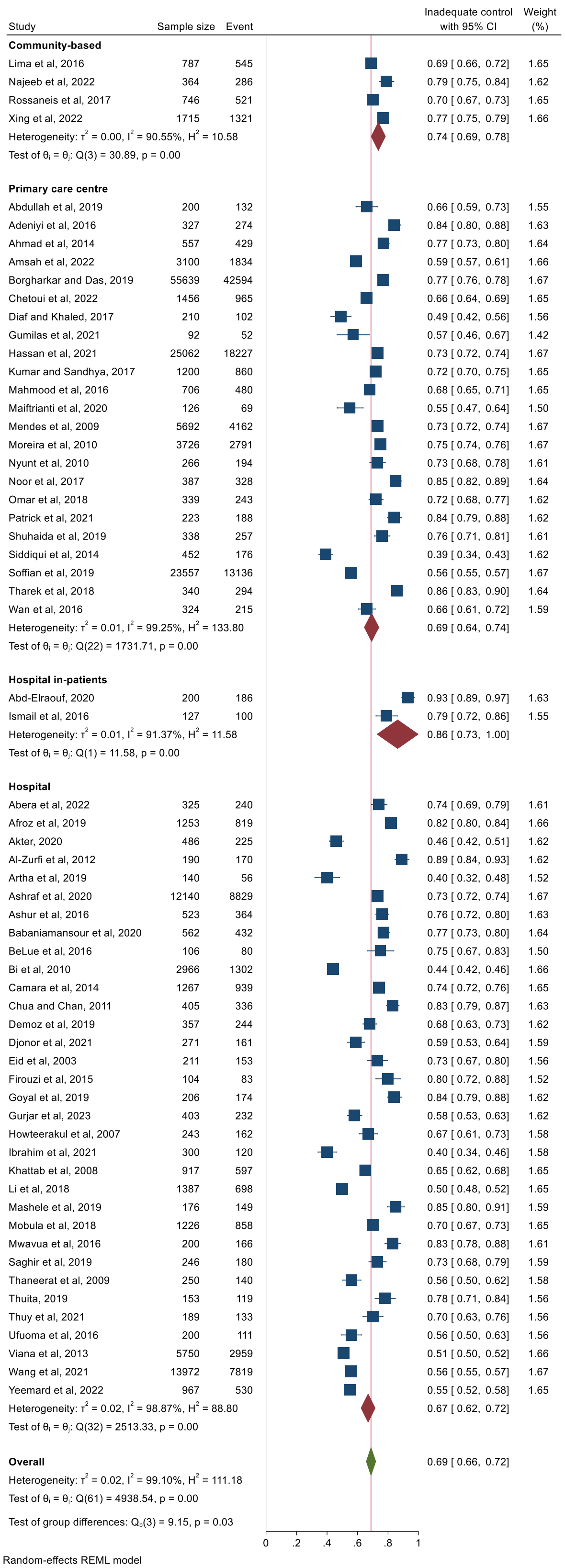

Supplementary figure 3: Pooled prevalence of inadequate glycaemic control (defined as HbA1c ≥6.5%, ≥7%, or ≥8%) stratified by study setting

Supplementary Table 1: Subgroup analysis for inadequate glycaemic control (HbA1c ≥6.5% or ≥7% or ≥8%) by socio-demographic, behavioural, anthropometric and clinical variables

Study characteristics	No of studies	Pooled proportion of inadequate glycaemic control (95% CI), p-value	I^2	Egger test (p-value)
**Socio-demographics**				
**Gender**	46			
Male		68 (64-72), <0.001	98.72%	0.012
Female		69 (65-73), <0.001	98.91%	0.044
**Education level**				
Below secondary	27	68 (63-74), <0.001	97.97%	0.505
Above secondary	22	66 (58-74), <0.001	94.46%	0.146
**Residence**				
Urban	13	68 (62-75), <0.001	95.71%	0.501
Rural	12	69 (60-77), <0.001	96.19%	0.954
**Behavioural**				
**Smoking**	13			
Yes		74 (68-79), <0.001	86.08%	0.342
No		67 (59-76), <0.001	98.57%	0.413
**Physical activity**	18			
Yes		63 (54-72), <0.001	97.63%	0.299
No		73 (67-79), <0.001	95.25%	0.642
**Dietary adherence**				
Yes	9	63 (51-74), <0.001	97.85%	0.074
No	8	78 (73-83), <0.001	87.03%	0.992
**Medication adherence**	15			
Yes		67 (59-74), <0.001	96.81%	0.344
No		76 (71-82), <0.001	89.17%	0.148
**Anthropometric**				
**Body Mass Index**				
Underweight	8	66 (53-78), <0.001	90.21%	0.664
Normal	26	63 (56-70), <0.001	99.02%	0.069
Overweight	26	66 (60-71), <0.001	98.74%	0.023
Obese	13	71 (63-78), <0.001	99.19%	0.537
**Clinical**				
**Duration of diabetes**	31			
<10 years		63 (57-69), <0.001	99.17%	0.757
≥10 years		74 (69-78), <0.001	98.05%	0.461
**Diabetes treatment**				
Diet	10	45 (27-62), <0.001	98.23%	0.462
Monotherapy	25	59 (51-67), <0.001	99.10%	0.026
Combination of OADs	12	72 (63-81), <0.001	98.46%	0.912

**Study characteristics**	**No of studies**	**Pooled proportion of inadequate glycaemic control (95% CI), p-value**	I^2	**Egger test (p-value)**
OAD and insulin	26	81 (76-86), <0.001	98.08%	0.351
Insulin only	21	76 (70-83), <0.001	96.44%	0.459
**Diabetes complications**				
Nephropathy	11	67 (59-76), <0.001	98.39%	0.413
Retinopathy	10	74 (66-82), <0.001	96.81%	0.709
Neuropathy	7	72 (61-86), <0.001	99.14%	0.895
CAD	7	65 (53-78), <0.001	98.70%	0.519
**Hypertension**				
Yes	20	66 (60-71), <0.001	99.12%	0.071
No	17	69 (62-75), <0.001	98.67%	0.232
**Dyslipidaemia**				
Yes	12	68 (61-74), <0.001	97.67%	0.126
No	10	64 (54-73), <0.001	98.06%	0.752

* OAD: Oral anti-diabetic, CAD: Coronary Artery Disease
